# Towards the preparation of synthetic outer membrane vesicle models with micromolar affinity to wheat germ agglutinin using a dialkyl thioglycoside

**DOI:** 10.3762/bjoc.15.90

**Published:** 2019-04-17

**Authors:** Dimitri Fayolle, Nathalie Berthet, Bastien Doumeche, Olivier Renaudet, Peter Strazewski, Michele Fiore

**Affiliations:** 1 Institut de Chimie et Biochimie Moléculaires et Supramoléculaires, Université de Lyon, Claude Bernard Lyon 1, 43 blvd du 11 novembre 1918, 69622 Villeurbanne Cedex, France; 2 Univ. Grenoble Alpes, CNRS, DCM UMR 5250, F-38000 Grenoble, France

**Keywords:** glycolipids, outer membrane vesicles, synthetic vaccines

## Abstract

A series of alkyl thioglycosides and alkyl thiodiglycosides bearing glucose and *N*-acetylglucosamine residues were prepared by thiol–ene coupling in moderate to good yields (40–85%). Their binding ability towards wheat germ agglutinin was measured by competitive enzyme-linked lectin assays. One of the synthetic compounds presenting two GlcNAc units showed the highest inhibitory effect of this study with an IC_50_ of 11 µM corresponding to a 3182-fold improvement compared to GlcNAc. These synthetic molecules were used to produce giant vesicles, alone or in mixture with phospholipids, mimicking bacterial outer membrane vesicles (OMV) with potential antiadhesive properties.

## Introduction

Outer membrane vesicles (OMV) [1], lipid bilayer vesicles released from the outer membrane of Gram-negative bacteria, are considered today as attractive candidates for vaccine delivery. However, they have some disadvantages, they are not easy to produce and are difficult to characterize [2–3]. Moreover, toxic lipopolysaccharides (LPS) present in the outer membrane of most of Gram-negative bacteria prevent the medical use of OMV from natural sources [4]. Licensed vaccines based on crude OMV are currently available to contribute to the prevention and to control at least twenty-five infections including pulmonary ones [5]. Developing synthetic vaccines against cancer [6–7] or Gram-negative bacteria [7] are challenges for the current research in the field. Artificial OMV, composed of synthetic and non-toxic, non-immunogenic phospholipids and glycolipids are good candidates for drug or vaccine delivery. One of the most common reactions used to prepare monoalkyl glycosides is the Fisher reaction between pyranoses and fatty alcohols of different lengths [8–9]. Alkyl thioglycosides are known for their properties as co-surfactants [10] and present interesting antimicrobial activities [11], acting as glycosidase inhibitors and being resistant towards glycoside hydrolases [12–13]. Some of them have been obtained by coupling protected glycosyl thiolates and *n*-alkyl halides [14–16]. Moreover, mechanochemical thioglycosylation of glycosyl acetates was used for the synthesis of *n*-alkyl 1-thio-α-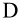-glycosides as carbohydrate mesogens [17]. Unfortunately, the preparation of alkyl glycosides cannot be carried out with unprotected thio-glycosides, implying orthogonal protection and deprotection steps in order to obtain unprotected glycolipids. We decided to investigate the formation of artificial OMV composed of long chain alkyl thioglycosides synthesized by the photoinduced radical addition of thiols to alkenes – known as thiol–ene coupling, TEC – a very efficient metal-free click reaction [18–19]. We prepared *n*-alkyl 1-thio-β-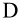-glycosides from unprotected sugar anomeric thiols, commercial *n*-alkenes and one synthetic lipophilic scaffold presenting two reactive alkenyl ends. Although thiyl-radical-mediated reactions have been extensively investigated for the preparation of carbohydrate derivatives [20] and some dithioether phospholipid and glycolipid analogues [21–22], no examples were reported for the synthesis of *n*-alkyl thioglycosides by using thiol–ene coupling [18–19]. In addition to its high efficiency and selectivity, the TEC reaction does not require any metal, a key feature for the preparation of potential vaccines.

## Results and Discussion

### Preparation of alkyl glycosides from unprotected sugars and lipophilic scaffolds

**Figure 1:**
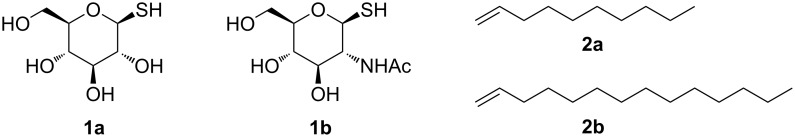
Structure of the β-thiols **1a** and **1b** and of the commercial alkenes **2a** and **2b**.

**Scheme 1:**
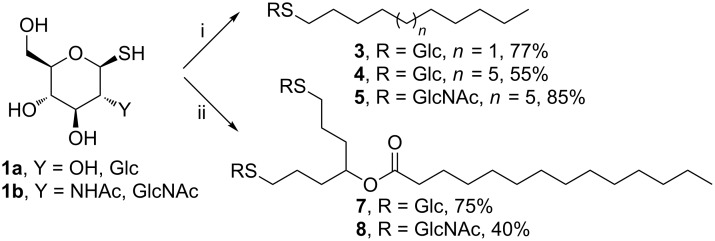
Synthesis of the *n*-alkyl thioglycosides **3**–**5**, **7** and **8**. Detailed reaction conditions are reported in the experimental part; i) **1a** or **1b** (3 equiv), **2a** or **2b** (1 equiv), DPAP (0.3 equiv); ii) **1a** or **1b** (3 equiv), **6** (1 equiv), DPAP (0.6 equiv). TECs were performed at room temperature under UV light (λ_max_ 365 nm) for 30 min (with **2a** and **2b**) or 60 min (with **6**) in methanol (**2a**) or DMF (**2b** and **6**); DPAP = 2,2-dimethoxy-2-phenylacetophenone.

Thioglycolipids are not native in OMV [1], but present several advantages compared to natural and synthetic glycolipids, linked by a chemically and enzymatically non-stable acetal function. Owing to their straightforward access, and the stability of thioether conjugates, 1-thio-β-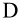-glucopyranose (**1a**, Figure 1), and 2-acetamido-2-deoxy-1-thio-β-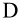-glucopyranose (**1b**, Figure 1) have been selected for this study and were prepared as previously reported [23]. Commercially available 1-decene (**2a**) and 1-tetradecene (**2b**) were selected for the synthesis of *n*-alkyl 1-thio-β-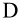-glycosides (**3**–**5**, Scheme 1), under UV-A irradiation (λ_max_ 365 nm), in the presence of the photoinitiator 2,2-dimethoxy-2-phenylacetophenone (DPAP).

**Scheme 2:**
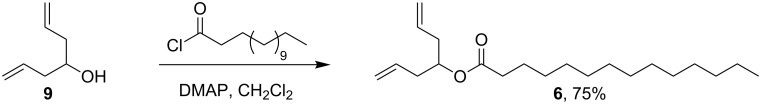
Synthesis of the lipophilic scaffold **6**; DMAP = *N,N*-dimethylaminopyridine.

The lipophilic scaffold hepta-1,6-dien-4-yl tetradecanotate (**6**, Scheme 2) was obtained from hepta-1,6-dien-4-ol and served for the preparation of compounds **7** and **8**. Hepta-1,6-dien-4-ol was prepared according to experimental procedures previously reported by others [24–25], then the myristoyl group (C_14:0_) was added using myristoyl chloride in the presence of DMAP (Scheme 1) [26].

### NMR monitoring

#### Following the formation of thioglycosides by ^1^H NMR analysis

**Figure 2:**
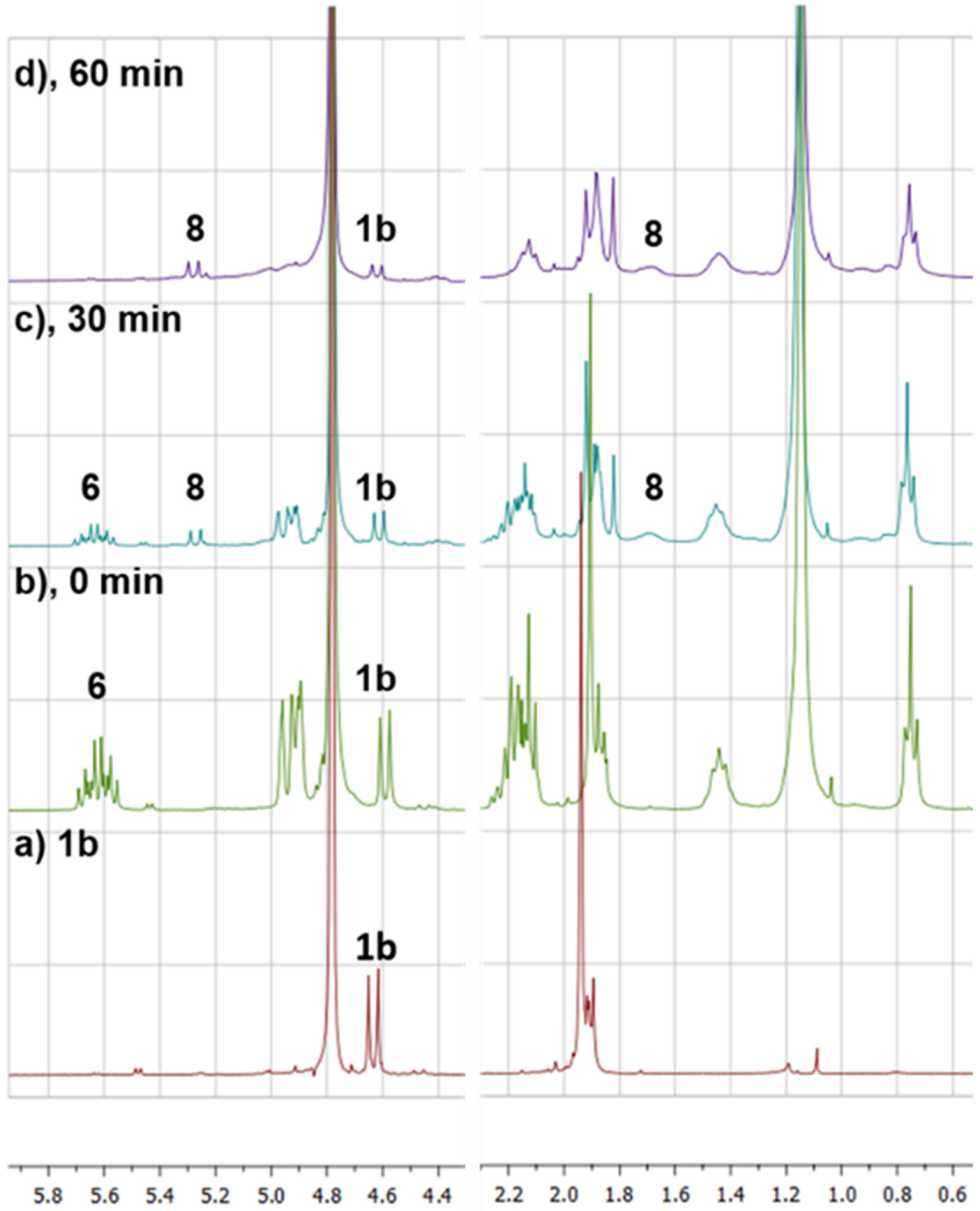
Periodic monitoring by ^1^H NMR (300 MHz, DMF-*d*_7_) of the formation of product **8** from a mixture compounds **1b** and **6**. a) Spectrum of pure **1b**; b) through d) crude reaction mixture after 0, 30 and 60 minutes of irradiation, respectively.

A test reaction, based on 10 mg of **1a**, was performed in deuterated methanol (MeOD) and the formation of compound **3** was monitored by periodical ^1^H NMR experiments, as illustrated in Supporting Information File 1, Figure S1. The signal corresponding to the double bond of **2a** (δ_H_ = 5.80–5.60 ppm) disappeared together with the signals of the α-methylene of **2a** (δ_H_ = 1.96 ppm). The appearance of a new doublet at 4.24 ppm with a coupling constant (*J* = 9.7 Hz) typical for an anomeric proton with β configuration, together with a new signal at 2.60 ppm corresponding to the α-methylene of the newly formed thioacetal bond, indicate the formation of **3**. The aromatic protons of DPAP (δ_H_ = 8.00 and 7.20 ppm, not shown) and the aliphatic chain signals (δ_H_ = 1.30 and 0.75 ppm) did not shift during the irradiation time. The product was not isolated and the reaction was repeated on a 50 mg scale in methanol, giving **3** in good yields (75%). The poor solubility of **2b** in methanol prompted us to use *N*,*N*-dimethylformamide (DMF) for the synthesis of **4** and **5**. Compounds **4** and **5** were isolated in good yields (55 and 75%, respectively) [27]. The syntheses of compounds **7** and **8** were carried out in DMF as well. As substantial amounts of **6** were isolated after the reaction, indicating an incomplete conversion to **8** (53%), the course of the reaction was followed by ^1^H NMR in deuterated DMF (Figure 2). The formation of **8** occurred with a progressive disappearing of the signals of the alkenyl group of 6 (δ_H_= 5.70–5.55 ppm) in one hour. The anomeric proton of the thiol **1b** (δ_H_ = 4.63 ppm) progressively decreased in intensity with apparition of new signals corresponding to the new anomeric protons (δ_H_ = 5.26 ppm) and the α-CH_2_ of the thioether bond (δ_H_ = 1.70 ppm). Compound **8** was isolated by column chromatography in 75% yield and no traces of starting material **6** were found. In addition, no intramolecular addition products neither mono-glycosylated compounds were observed.

### Preparation of vesicles upon hydration of a thin film of phospholipids and glycolipids

**Figure 3:**
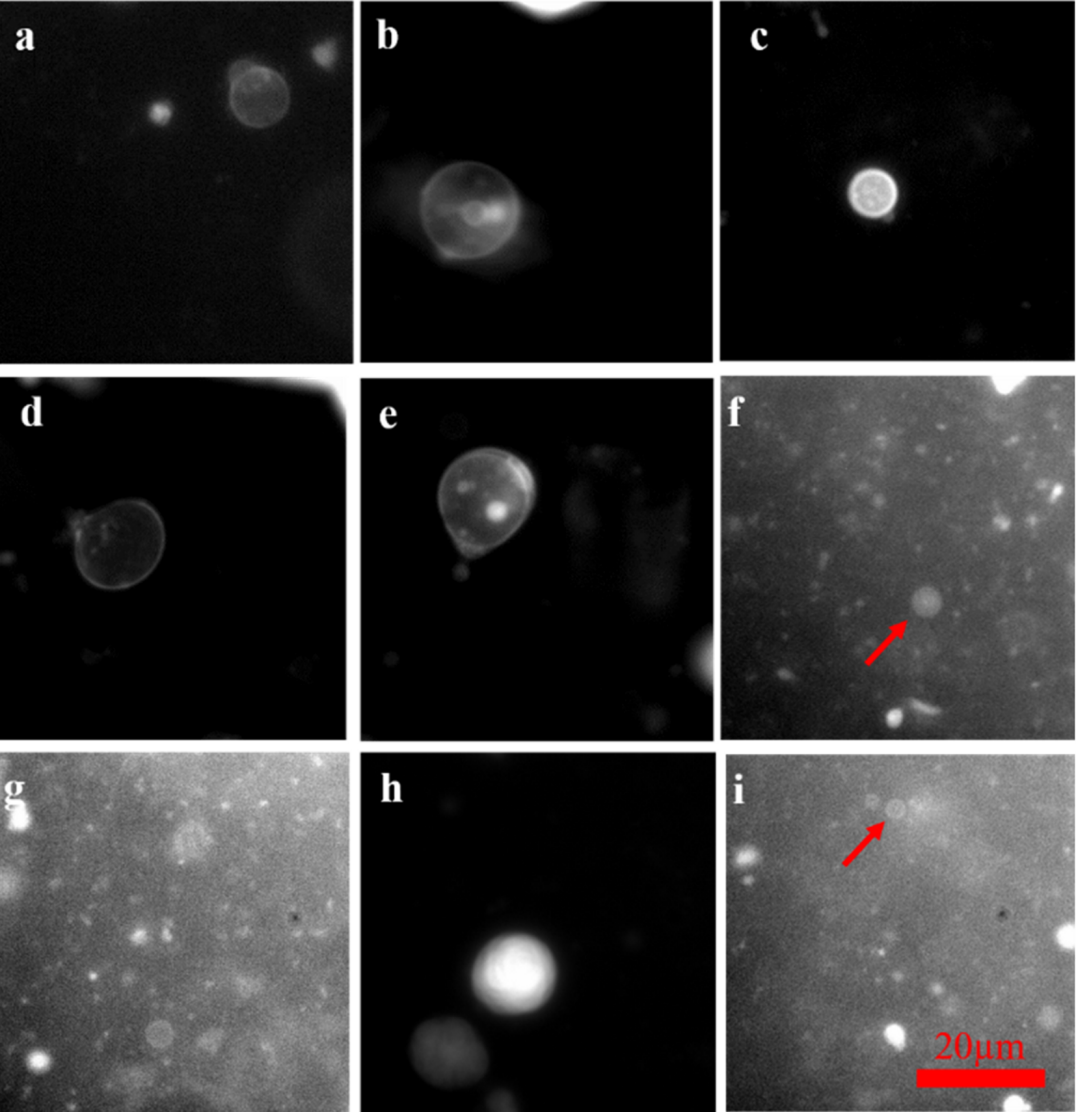
Micrographs of giant vesicles and lipid aggregates obtained from the gentle hydration (in PBS, pH 7.4) of compounds **8** and **5**, alone and in binary and ternary mixtures with POPC. **a**–**e**) POPC/**8**, 9:1 molar ratio; f and g) pure **8**; h) **5**/**8**, 1:1 molar ratio; i) pure **5**; All the samples were prepared with 1 mM lipids and stained with NileRed^®^ (1 mM in EtOH, 1 µL, λ_[excit]_ = 561 nm) before the microscopic observation. Red arrows are used to indicate small GVs. The scale bar is 20 µm for all micrographs in this figure.

Once synthesized, the compounds **5** and **8** bearing GlcNAc residues were used to produce giant vesicles (GVs) upon hydration with PBS (pH 7.4) by modifying reported procedures [28–30]. GVs were prepared by hydrating a thin film containing 90:10 (mol %) of those glycoconjugates in mixture with a natural phospholipid: 1-palmitoyl-2-oleoyl-*sn*-glycero-3-phosphocholine (POPC). As expected, the mixture once hydrated showed a high tendency to vesiculate (Figure 3, images a–e and h). Moreover, a thin layer of compounds **5** or **8** without POPC, hydrated in the same conditions, gave similar giant vesicles (Figure 3, images f,g and i). Microscopic observation suggested that those supramolecular assemblies microscopically resemble the well-known outer membrane vesicles (OMV) [1]. The tentative dissolution of compounds **5***–***8** in PBS (pH 7.4) produced a slightly turbid suspension, suggesting that those molecules formed a mixture of microscopically visible vesicles (diameter >0.5 µm), small vesicles (diameter <0.5 µm) and perhaps micelles (diameter in the nanometer range). However, due to our interest to study the interaction of OMV-like objects [1] we prepared giant vesicles resembling the OMV by using mixtures of fully synthetic compounds and natural occurring phospholipids. The vesicles showed stability over time; however, osmotic stress, sudden temperature change and accidental drying of the hosting solution inevitably cause the disruption of the supramolecular assembly [23]. Analyses under saline stress were not performed as the ultimate aim is to use of those OMV models under physiological conditions.

### Biological evaluation of the synthesized compounds

**Figure 4:**
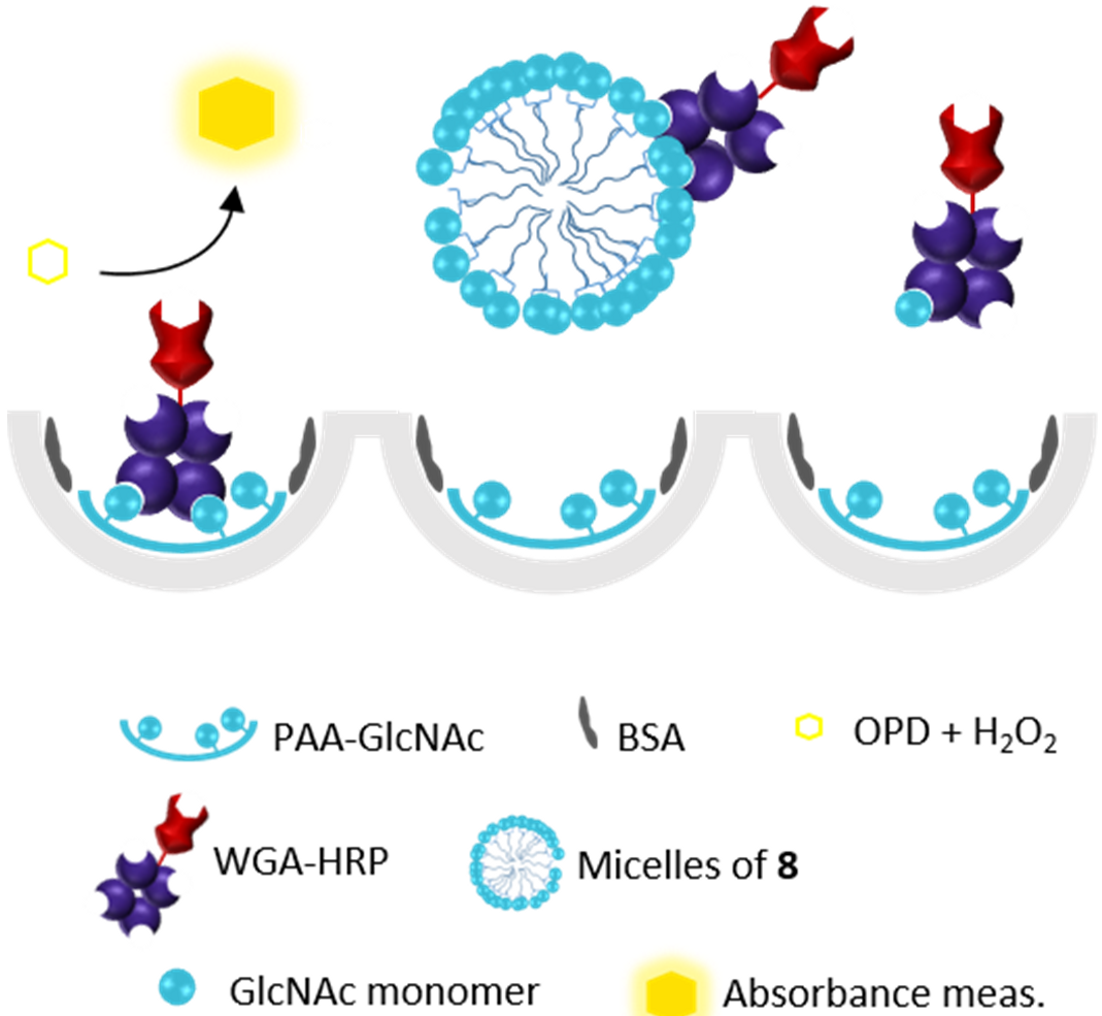
A simplified (and not in scale) representation of the ELLA assay, to study the interaction between GVs or micelles of compound **8** or **5** and WGA. OPD: *O*-phenylenediamine dihydrochloride; PAA: poly[*N*-(2-hydroxyethyl)acrylamide].

Competitive enzyme-linked lectin assays (ELLA) were used to evaluate the binding properties of compounds **5** and **8** towards wheat germ agglutinin (WGA), a lectin from *Triticum vulgaris* that is specific for *N*-acetylneuraminic acid and *N*-acetylglucosamine. This assay consists in measuring the ability of the compounds to inhibit the binding of WGA horseradish peroxidase-labelled (WGA-HRP) to a GlcNAc-polyacrylamide conjugate following the procedure described in Figure 4.

WGA is a homodimer in which each monomer is organized into four domains (A–D) containing adjacent “primary” (B and C) and “secondary” (A and D) domains binding sites with different affinities to GlcNAc [31]. The proximity of adjacent binding sites (≈14 Å) [32] makes this lectin an excellent candidate to investigate multivalent carbohydrate–protein interactions [33]. Although the scientific literature is rich of remarkable examples of biologically active carbohydrate-based compounds with high affinity to lectins and with antibiotic activities [34], lectin recognition by *n*-alkyl thioglycoside liposomes remains unprecedented [33–34]. Moreover, the calculated distance between the two sugar moieties of compound **8** (up to 13 Å, Figure 6) and the presence of flexible arms make these compounds similar to other efficient multivalent glycoconjugates obtained by TEC of sugar thiols with different multivalent scaffolds such as octasilsesquioxanes [35], cyclopeptides [36–37] and polymers [38].

**Figure 5:**
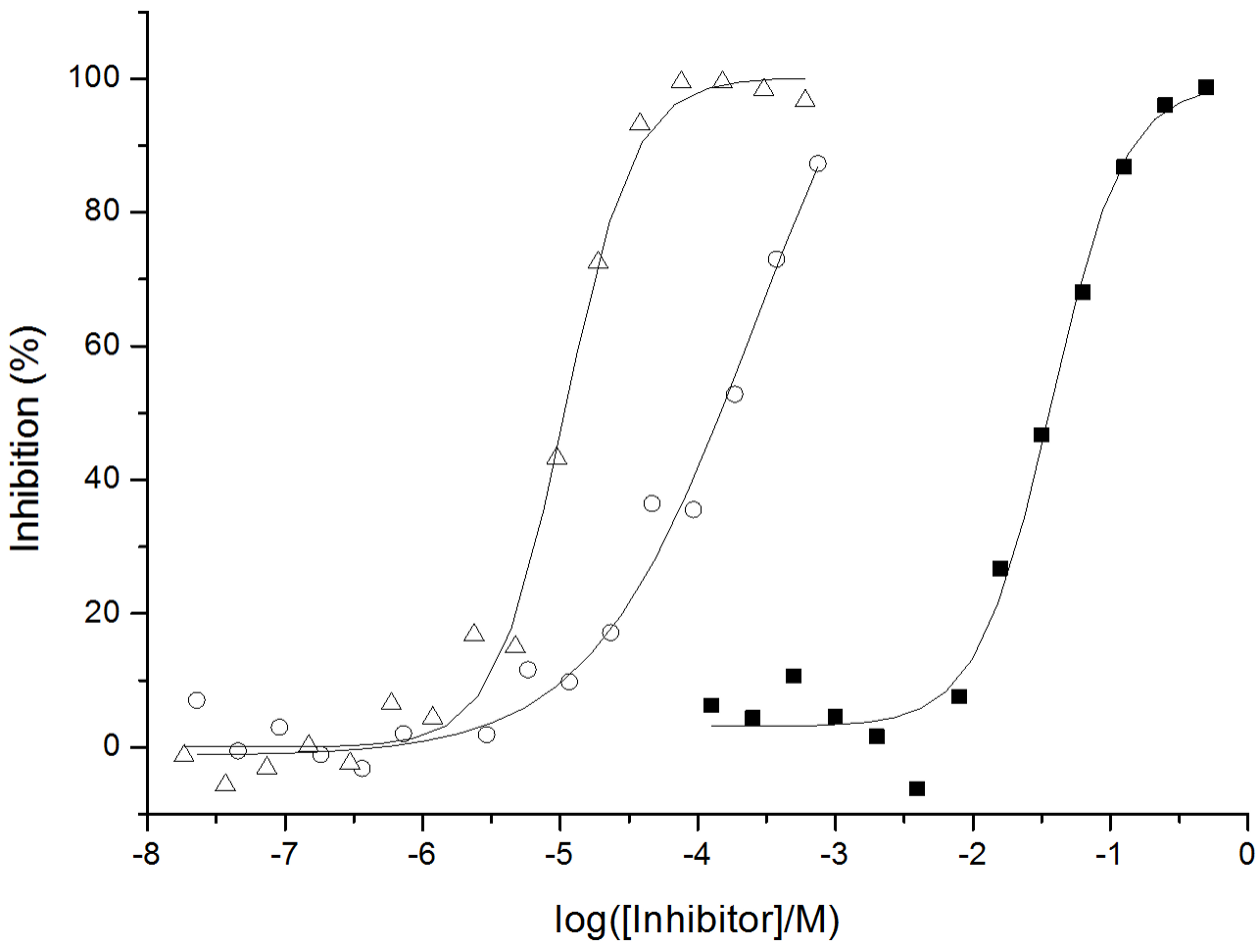
Inhibition curves for the binding of WGA-HRP to PAA-GlcNAc by D-GlcNAc The symbols (■), (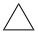) and (○) represent the monomer (GlcNAc) and the lipophilic thioglycoconjugates **8** and **5**, respectively.

Both compounds **5** and **8** showed low IC_50_ values (a tenth to some hundreds of micromolars) where the GlcNAc monomer displays only 35 mM (Table 1). Compounds **4** and **7** bearing one and two glucose residues served as negative controls as WGA has no affinity towards glucose. This result clearly shows the high potential of the lipophilic *n*-alkyl thioglycoconjugates for lectine binding. In particular, compound **8** showed the highest inhibitory effect of this study with an IC_50_ of 11 µM corresponding to a 3000-fold improvement compared to the monomer control. This result suggests a higher participation of sugar units in the lectin binding for this compound.

**Table 1:** Inhibition of the adhesion of WGA-HRP to GlcNAc-coated microtiter plates as determined by ELLA^a^.

Compound	IC_50_ (μM)	rp^b^

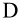 -GlcNAc	35 000	1
**5**	142	246
**8**	11	3182

^a^Experiments were performed in triplicate except for compound **5. **^ b^rp: relative potency = IC_50_(lipophilic thioglycoconjugate)/IC_50_(GlcNAc).

### Docking calculation for model glycolipids

To appreciate the possible binding of **4**, **5**, **7** and **8** onto WGA, a docking simulation was performed using the crystal structure of WGA obtained in the presence of GlcNAc (PDB 2UVO) and of other GlcNAc derivatives (PDB 2X52 and 4AML). The docking experiments were performed using analogous molecules bearing acetate and butanoate alkyl chains (namely **4C_2_**, **4C_4_**, **5C_2_**, **5C_4_**, **7C_2_**, **7C_4_**, **8C_2_** and **8C_4_** according to the alkyl length, see Figures S5 and S6 in Supporting Information File 1) instead of the long alkyl chains, for calculation simplicity.

The docking protocol was validated using GlcNAc and Glc as ligands. In such a case, the poses found with GlcNAc were strictly identical to the ones observed in the crystal structure while no poses were obtained using Glc as ligand. Docking experiments using Glc derivatives **4C_2_**, **4C_4_**, **7C_2_** and **7C_4_** do not lead to any poses, suggesting, as observed in the ELLA experiments, that WGA do not bind them.

**Figure 6:**
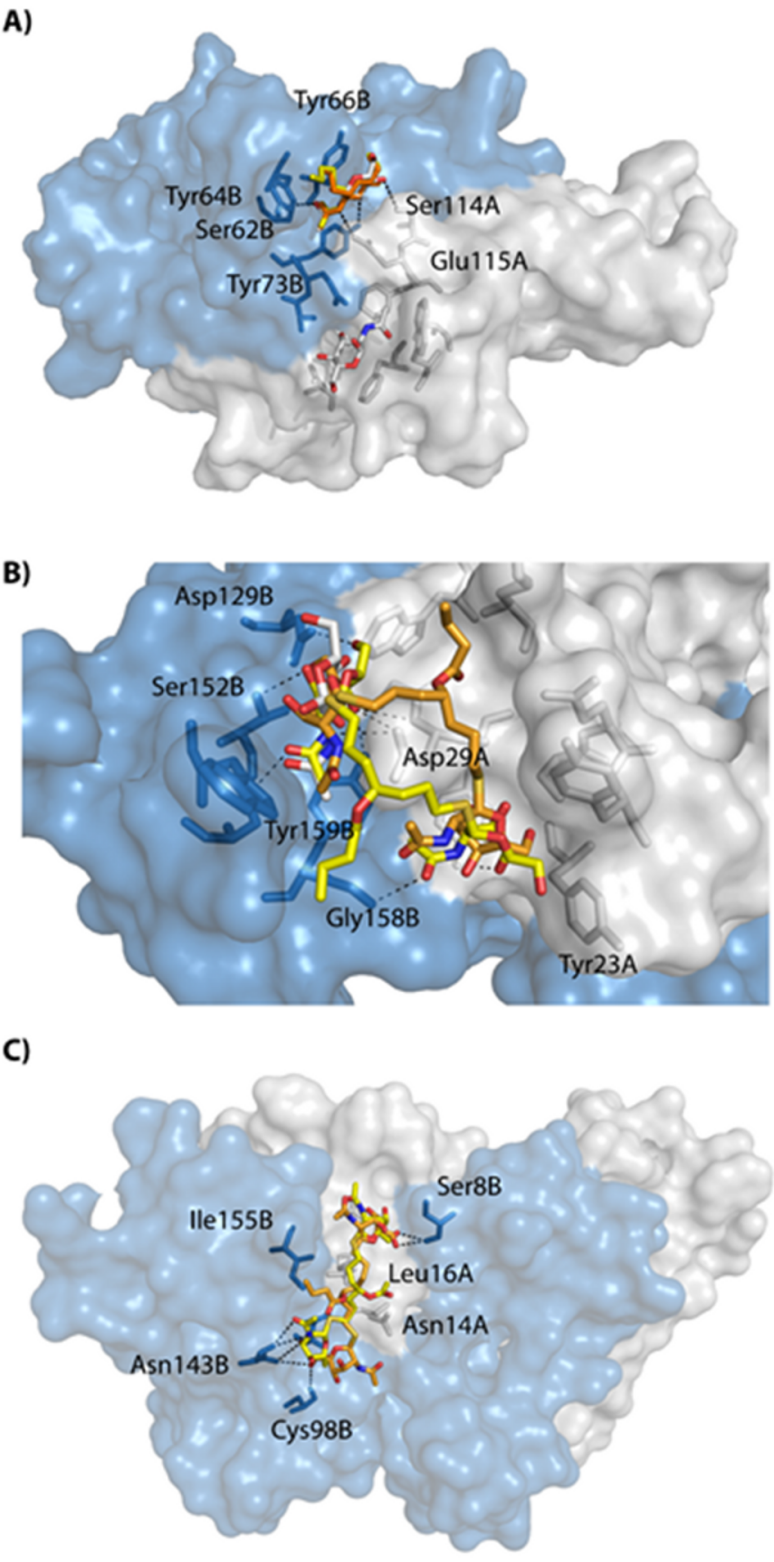
Main poses obtained from docking experiments. WGA (PDB 2UVO) surface is shown in white for monomer A and in blue for monomer B. Residues within 4 Å of the docked compound are in white stick representation. A) Representative poses obtained for **5C_2_** (yellow sticks) and **5C_4_** (orange sticks) at the B1C2 primary site superimposed with co-crystallized GlcNAc (white sticks). B) Representative poses of **8C_4_** (orange and yellow sticks) obtained at the D2A1 and A2 binding superimposed with co-crystalized GlcNAc (white sticks). C) Representative poses of **8C_2_** (yellow sticks) and **8C_4_** (orange sticks) in the cleft between the two WGA monomers.

About half of the poses obtained from compounds **5C_2_** and **5C_4_** (bearing a single sugar moiety) were found on the primary (BC) and secondary (AD) binding sites with energies ranging from −4.5 to −4.9 kcal mol^−1^ (pose A, Figure 6 and Supporting Information File 1, Figure S5) [31–32,39–40]. The binding of **8C_2_** and **8C_4_** onto WGA lead to two main poses. The first one (energies ranging from −5.3 to −6.2 kcal mol^−1^) corresponds to the binding of both GlcNAc moieties onto the secondary binding sites D2A1 and A2 (pose B, Figure 6). None of the poses were found to occupy primary binding sites as it was observed for **5C_2_** and **5C_4_**. The first GlcNAc moiety of **8C_2_** and **8C_4_** interacts with Asp29A, Asp129B, Ser148B and Tyr159B and nearly superimposes with GlcNAc in the WGA crystalline structure (site D2A1). The second GlcNAc moiety is at the vicinity of Ser19A and Tyr30A residues (A2 binding site), therefore mimicking the binding of two GlcNAc molecules on WGA. The distance between the two GlcNAc residues is 11–12 Å [41]. The alkyl chains (acetyl or butyl) fit in the non-polar environment provided by Trp150B and by Gly158B or by the acyl moiety of Lys33A (pose B, Figure 6). Nevertheless, there is no obvious involvement of the ester bond in the binding of **8C_2_** or **8C_4_** at these secondary sites. The other main poses of **8C_2_** and **8C_4_** present the ligands adopting a linear conformation in a cleft between both monomers of WGA with similar energies (−5.3 to −6.2 kcal mol^−1^, pose C, Figure 6 and Figure S6 in Supporting Information File 1). This cleft is defined by neutral residues Asn14A and Asn101B but also by the non-polar Leu16A and Ile155B that provide hydrophobic environment for the heptanol moiety in **8C_2_** and **8C_4_**. This region of the protein also lacks charged residues that would prevent **8C_2_** or **8C_4_** from binding. GlcNAc moieties are proposed to interact with the hydroxy group of Ser8B and the carbonyl of Cys24A from one side and with Asn143B, the carbonyl of Cys98B or Asn14A on the other side. The alkyl chains of **8C_2_** and **8C_4_** are found close to Ile155B and head out of the cleft if a longer alkyl chain is used (as in **8**). These observations suggest that the herein synthesized bidentate analogues of GlcNAc would interact with WGA differently than compounds bearing one sugar residue. The short distance between the two sugar moieties as well as the presence of the non-polar alkyl chains seems to prevent the binding to primary sites.

## Conclusion

A series of glucose and *N*-acetyl glucosamine-based thioglycolipids were synthesized for the first time by using thiol–ene coupling in the absence of protecting groups and in good yields. Monosaccharides where selected on the basis of similar researches carried out in the field [38]. We reported a simplified access to *n*-alkyl thioglycosides as building blocks for the preparation of OMV models. The obtained *n*-alkyl thioglycosides were tested for the first time for lectin recognition and showed promising results, as their aggregates in PBS displayed micromolar affinity with WGA. In addition, we produced multilamellar giant vesicles of variable size from the glycolipids alone or in mixture with phospholipids. The vesicles we obtained showed similarities with OMV structures observed by Holst and co-workers (cf. Supporting Information File 1, Figure S4) [1]. This work suggests that mixtures of phospholipids such as POPC and small amounts of bioactive glycolipids should give a controlled bilayer. Such a construct could represent attractive carriers for vaccines once the sugar moiety of the glycolipid is substituted by *T*_n_ or *T*_f_ antigens [42–43] or using natural glycolipids from Gram-negative bacteria such as lipid A [4]. This work represents the first step towards the formulation of an heterogeneous, stable systems (under biological conditions) that can be used for several biological purposes including synthetic vaccines, supramolecular adjuvants, glycolipid–phospholipid drug delivery systems and for the formulation of GVs that can be used as tools to bind to various bacterial lectins depending on the mono- or disaccharides used. As a first and relevant conclusion, in silico and in vitro studies demonstrated that two of those compounds, bearing one or two *N*-acetyl glucosamine moieties are capable to bind strongly to the lectin WGA.

## Experimental

### Materials and methods

Myristoyl chloride was from TCI-Europe (Antwerp, Belgium) and used without further purification. 1-Palmitoyl-2-oleoyl-*sn*-glycero-3-phosphate (POPC) was from Avanti Polar Lipids (Alabaster, AL, USA). 2,3,4,6-Tetra-*O*-acetyl-α-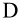-glucopyranosyl bromide and *N*-acetyl-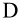-glucosamine were from Carbosynth (San Diego, CA, USA). Wheat germ agglutinin (WGA), horseradish peroxidase-labelled WGA (WGA-HRP), bovine serum albumin (BSA) and SIGMA FAST *O*-phenylenediamine dihydrochloride (OPD) were from Sigma-Aldrich. Polymeric *N*-acetyl-β-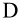-glucosamine (PAA-GlcNAc) was from Lectinity Holding, Inc., Moscow. DPAP (2,2-dimethoxy-2-phenylacetophenone) and all the other reagents were from Sigma-Aldrich (Merck KGaA, Darmstadt, Germany). Reaction solvents were from Fischer Scientific (Illkirch, France). Deuterated solvents for NMR were from Euriso-top (France). All sensitive reactions were carried out in oven-dried glassware under an inert argon atmosphere. Room temperature (rt) refers to 20–25 °C. All thiol–ene couplings were performed with a Philips UVA lamp irradiating at 365 nm (Mgc typ838 150 W, 6 tubes). Thin-layer chromatography (TLC) was carried out on aluminum sheets coated with silica gel 60 F254 (Merck, Kenilworth, USA). TLC plates were inspected by UV light (λ = 254 nm) and developed by treatment with a mixture of 10% H_2_SO_4_ in EtOH/H_2_O (1:1 v/v), followed by heating. Electrospray-ionization mass spectra (ESIMS) were recorded using a Bruker Q-Tof Micromass spectrometer. NMR spectra were recorded in DMSO-*d*_6_, CDCl_3_, CD_3_OD or DMF-*d*_7_ on a Bruker Avance 300 spectrometer at 300 and 75 MHz and on a Bruker Avance 400 at 400 and 100 MHz for ^1^H, and ^13^C, respectively. The attached proton test experiment (APT) was performed to replace the ^13^C experiment when required. Chemical shifts of solvents (CDCl_3_: δ_H_ = 7.26 and δ_C_ = 77.13; CD_3_OD: δ_H_ = 3.31 and δ_C_ = 49.50; DMF-*d*_7_ δ_H_ = 8.01, and δ_C_ = 163.15) served as internal references. Signal shapes and multiplicities are abbreviated as br (broad), s (singlet), d (doublet), t (triplet), q (quartet), quint (quintet) and m (multiplet). Where possible, a scalar coupling constant *J* is given in hertz (Hz). Optical rotations were measured as CHCl_3_ solutions on a JASCO P-1010 digital polarimeter and converted to specific rotations [α]_D_. Micrographs were recorded with an inverted microscope (Zeiss LSM 800) equipped with a 50× objective through the AxioCam software. Micrographs were used without any graphical treatment and the image size was adjusted respecting the *x*/*y* pixel proportions

### Synthesis of compounds **3–8**

**Compound 3:** A stirred solution of **1a** (47 mg, 0.24 mmol, 3 equiv), **2a** (11.2 mg, 0.08 mmol, 1 equiv) and DPAP (7.71 mg, 0.024 mmol, 0.3 equiv) in MeOH (660 μL) was irradiated at room temperature for 30 min and then concentrated. The residue was purified by silica flash chromatography (gradient: 100:0–80:20 CHCl_3_/MeOH v/v). Yield: 77% (20.7 mg, 0.06 mmol); [α]_D_^25^ +11.8 (*c* 0.1, CHCl_3_); ^1^H NMR (300 MHz, CDCl_3_) δ_H_ 5.35 (d, *J* = 4.6 Hz, 1H), 4.42 (d, *J* = 9.4 Hz, 2H), 3.83 (s, 2H), 3.56 (dd, *J* = 7.9 Hz, 2H), 3.44 (s, 1H), 3.36 (dd, *J* = 8.9 Hz, 2H), 2.67 (t, *J* = 7.2 Hz, 2H), 1.58 (dt, *J* = 7.4 Hz, 2H), 1.23 (s, 12H), 0.85 (t, *J* = 6.7 Hz, 3H); ^13^C NMR (75 MHz, CDCl_3_) δ_C_ 14.3, 22.9, 29.2, 29.4, 29.6, 29.9, 30.3, 31.1, 61.7, 69.5, 72.9, 78.0, 79.7, 86.4; HRMS (*m/z*): [M + H]^+^ calcd for C_16_H_32_O_5_S, 336.487; found, 336.493.

**Compound 4:** A stirred solution of **1a**, (50 mg, 0.25 mmol, 3 equiv), **2b** (16.4 mg, 21 µL, 0.085 mmol, 1 equiv) and DPAP (5.9 mg, 0.025 mmol, 0.3 equiv) in DMF (1 mL) was irradiated at room temperature for 30 min and then concentrated. The residue was purified by silica flash chromatography (gradient: 100:0–80:20 CHCl_3_/MeOH v/v). Yield: 55% (20.3 mg, 0.047 mmol). Chemical analyses are in agreement with what was previously reported [44].

**Compound 5:** A stirred solution of glycosyl thiol **1b** (49.8 mg, 0.21 mmol, 3 equiv), **2b** (13.7 mg, 0.07 mmol, 1 equiv) and DPAP (5.1 mg, 0.02 mmol, 0.3 equiv) in DMF (660 μL) was irradiated at room temperature for 30 min and then concentrated. The residue was purified by silica flash chromatography (gradient: 100:0–80:20 CHCl_3_/MeOH v/v). Yield: 85% (25.8 mg, 0.06 mmol); ^1^H NMR (400 MHz, MeOD) δ_H_ 4.46 (d, *J* = 12.0 Hz, 1H), 3.86 (dd, *J* = 12.0 Hz, 1H), 3.72 (t, *J* = 12.0 Hz, 1H), 3.70–3.64 (m, 2H), 3.32 (t, *J* = 12.0 Hz, 1H), 3.22 (t, *J* = 12.0 Hz, 1H, partially masked by HOD signal), 2.70 (m, 2H) 1.97 (s, 3H), 1.61 (m, 2H), 1.29 (s, 14H), 0.90 (t, *J* = 8.0 Hz, 3H); ^13^C NMR (100.0 MHz, MeOD) δ 12.9, 21.6, 22.3, 25.6, 28.6, 28.9, 29.1, 29.2, 29.3–29.4, 54.9, 61.4, 70.6, 76.0, 80.8, 84.3; [α]_D_^25^ +11.8 (*c* 0.1, CHCl_3_); HRMS (*m/z*): [M + H]^+^ calcd for C_22_H_44_NO_5_S, 434.2921; found, 434.2935.

**Compound 6:** Hepta-1,6-dien-4-ol [24–25] (500 mg, 4.5 mmol), myristoyl chloride (2.9 mL, 10.7 mmol) and DMAP (1.3 g, 10.7 mmol) were dissolved in 10 mL of dry CH_2_Cl_2_. The solution was stirred for 16 h, then transferred in a separation funnel and washed with saturated NaHCO_3_ (2 × 50 mL) and brine (50 mL), dried over Na_2_SO_4_ and concentrated. The crude mixture was purified on a column of silica (isocratic, CH_2_Cl_2_) from which **3** was obtained as a transparent oil. Yield: 78% (1.12 g, 3.50 mmol). ^1^H NMR (300 MHz, CDCl_3_) δ_H_ 5.80–5.66 (m, 2H), 5.08 (d, *J* = 7.4 Hz, 2H), 5.03 (br s, 2H), 4.95 (dd, *J* = 12.4, 5.9 Hz, 1H), 2.34–2.30 (m, 4H), 2.23 (t, *J* = 8.3 Hz, 2H), 1.63–1.57 (m, 2H), 1.25 (br s, 20H), 0.87 (t, *J* = 6.7 Hz, 3H); ^13^C NMR (75 MHz, CDCl_3_) δ_C_ 14.1, 22.6, 25.0, 29.1, 29.2, 29.3, 29.4, 29.5–29.7, 31.9, 34.5, 38.0, 71.9, 117.7, 133.6, 173.3; HRMS (*m/z*): [M + H]^+^ calcd for C_21_H_38_O_2_, 345.2769; found, 345.2764.

**Compound 7:** A stirred solution of **1a** (117.6 mg, 0.6 mmol, 6 equiv (3 × site), **6** (34.6 mg, 0.1 mmol, 1 equiv) and DPAP (15.0 mg, 0.06 mmol, 0.6 equiv) in DMF (660 μL) was irradiated at room temperature for 60 min and then concentrated. The residue was purified by silica flash chromatography (gradient: 100:0–80:20 CHCl_3_/MeOH v/v). Yield: 75% (51.5 mg, 0.07 mmol). ^1^H NMR (400 MHz, CD_3_OD) δ_H_ (4.46 (d, *J* = 10.3 Hz, 2H), 3.87 (dd, *J* = 12.0, 2.2 Hz, 2H), 3.73 (dd, *J* = 10.3, 6.2 Hz, 2H) 3.71–3.66 (m, 2H), 3.65–3.63 (m, 2H), 3.54 (t, *J* = 6.7 Hz, 2H), 3.45–3.40 (m, 2H), 3.29–3.25 (m, 4H), 2.78–2.61 (m, 2H), 2.04–1.55 (m, 2H) 1.66–1.48 (m, 2H), 1.27 (br s, 10H), 0.87 (t, *J* = 6.0 Hz, 3H); ^13^C NMR (100 MHz, CD_3_OD) δ_C_ 13.0, 22.3, 25.5, 28.9, 29.1, 29.2, 29.3, 29.4, 29.5, 31.7, 32.3, 33.3, 54.9, 61.6, 70.6, 76.0, 80.8, 84.3, 172.1; [α]_D_^25^ 0.37 (*c* 0.1, MeOH); HRMS (*m/z*): [M + Na]^+^ calcd for C_33_H_62_NaO_12_S_2_, 736.3502; found, 736.3508.

**Compound 8:** A stirred solution of **1b** (142.2 mg, 0.6 mmol), **6** (34.7 mg, 0.1 mmol, 1 equiv) and DPAP (15.0 mg, 0.06 mmol, 0.6 equiv) in DMF (660 μL) was irradiated at room temperature for 120 min and then concentrated. The residue was purified by silica flash chromatography (gradient: 100:0–75:25 CHCl_3_/MeOH v/v). Yield: 40% (31.8 mg, 0.04 mmol). ^1^H NMR (300 MHz, CD_3_OD) δ_H_ 5.34 (m, 4H), 5.08 (m, 1H), 4.34–4.21 (m, 2H), 3.71 (d, *J* = 4.0 Hz, 2H), 2.35–2.21 (m, 4H), 2.00 (d, *J* = 8.0 Hz, 4H), 1.61 (d, *J* = 4.0 Hz, 4H), 1.27 (d, *J* = 16.1 Hz, 34H), 0.87 (t, *J* = 8.0 Hz, 6H); ^13^C NMR (75 MHz, CDCl_3_) δ_C_ 22.7, 24.8, 24.9, 25.6, 27.0, 27.1, 27.2, 29.0–29.2, 29.3, 29.4, 29.7, 29.8, 31.9, 34.0, 34.3, 61.5, 62.0, 66.0, 72.1, 129.7, 130.0, 173.4, 173.8, 173.9; [α]_D_^25^ 0.31 (*c* 0.1, MeOH); HRMS (*m/z*) calcd for C_37_H_68_N_2_O_12_S_2_, 796,4214; found, 796,4222.

**Preparation and observation of giant vesicles:** Giant vesicles (GVs) were prepared by the natural swelling method [23]. Lipids (mixture of commercial POPC and *n*-alkyl thioglycosides **5** or **8**) were dissolved in methanol (typically, 2 mL) in a 10 mL round-bottom flask. The solvent was completely evaporated under reduced pressure using a rotatory evaporator. The resulting thin lipid film was further dried for 180 minutes at 1 mbar/25 °C, and then hydrated for 16 hours – without shaking – with the aqueous buffer, termed “I-solution” (composed of 200 mM sucrose in 50 mM of PBS buffer, pH 7.4) to obtain an overall 1–2 mM lipid concentration. The hydration temperature was 25 °C. Three volumes of the thus obtained GVs were diluted with one volume of an aqueous isotonic buffer solution termed “O-solution” (composed of 200 mM glucose in 50 mM of PBS buffer, pH 7.4) and centrifuged at 5,000 rpm for 10 minutes in a bench-top Eppendorf mini-centrifuge. GVs were pelleted down in the Eppendorf tube due to the density difference between the I-solution and the O-solution. The supernatant was carefully removed, and the pellet was re-suspended in 100 µL of fresh O-solution. Each sample was stained with a Nile Red^®^ solution (1 mM in DMSO, 1 µL) before microscopic observation.

**Enzyme-linked lectin assays:** 96-well microtiter Nunc-Immuno plates (Maxi-Sorp) were coated with PAA-GlcNAc (100 μL per well, diluted from a stock solution of 5 μg mL^−1^ in 50 mM carbonate buffer pH 9.6) for 1 h at 37 °C. The wells were then washed with T-PBS (3 × 100 μL per well, PBS pH 7.4 containing 0.05% (v/v) Tween 20). This washing procedure was repeated after each incubation step. The coated microtiter plates were then blocked with BSA in PBS (3% w/v, 1 h at 37 °C, 100 μL per well). Serial two-fold dilutions of each inhibitor were pre-incubated 1 h at 37 °C in PBS-DMSO (9:1 v/v, 60 μL per well) in the presence of WGA-HRP (60 μL) at the desired concentration. The above solutions (100 μL) were then transferred to the blocked microtiter plates which were incubated for 1 h at 37 °C. After incubation, the plates were washed with T-PBS (3 × 100 μL per well) and then the color was developed using OPD (100 μL per well, 0.4 mg mL^−1^ in 0.05 M phosphate-citrate buffer) and urea hydrogen peroxide (0.4 mg mL^−1^). The reaction was stopped after 10 min by adding H_2_SO_4_ (30% v/v, 50 μL per well) and the absorbance was measured at 490 nm. The percentage of inhibition was determined using the following equation, where A is absorbance. % Inhibition = [(A(no inhibitor) – A(with inhibitor)/A(no inhibitor)] × 100. The percent of inhibition was plotted against the logarithm of the concentration of the sugar derivatives. The sigmoidal curves were fitted and the concentration at 50% inhibition of WGA-HRP binding to PAA-GlcNAc coated plates was determined (IC_50_).

**Docking experiments:** WGA structure was obtained from the Protein Data Bank (2UVO) and unbound ligands (water, GlcNAc, glycerol and pyroglutamic acid) were removed. PDB files for compounds **4C_2_**, **4C_4_**, **5C_2_**, **5C_4_**, **7C_2_**, **7C_4_**, **8C_2_** and **8C_4_** were prepared using OpenBabel (version 2.4.1). Docking was performed using autodock vina implemented in the Samson software (version beta 0.7.0, NANO-D Inria, https://www.samson-connect.net). A grid (60 × 60 × 60 Å) including the whole protein was used and 20 poses leading to the lowest energies were kept as significant. Pictures were prepared using PyMOL (version 0.99rc6, DeLano Scientific, San Carlos, CA, 700).

## Supporting Information

File 1 Additional Figures S1–S6 and spectra of synthesized glycolipids and starting materials.
