# Supplementary material for: Towards the preparation of synthetic outer membrane vesicle models with micromolar affinity to wheat germ agglutinin using a dialkyl thioglycoside
Source: Beilstein J Org Chem. 2019 Apr 17;15:937–46. doi: 10.3762/bjoc.15.90 (PMC6541351; doi:10.3762/bjoc.15.90)
Supplement: File 1 — Additional Figures S1–S6 and spectra of synthesized glycolipids and starting materials. [file Beilstein_J_Org_Chem-15-937-s001.pdf]

BEILSTEIN JOURNAL OF ORGANIC CHEMISTRY

Supporting Information

for

Towards the preparation of synthetic outer membrane vesicle models with micromolar affinity to wheat germ agglutinin using a dialkyl thioglycoside

Dimitri Fayolle, Nathalie Berthet, Bastien Doumeche, Olivier Renaudet, Peter Strazewski and Michele Fiore

Beilstein J. Org. Chem. **2019,** *15,* 937–946. doi:10.3762/bjoc.15.90

Additional Figures S1–S6 and spectra of synthesized glycolipids and starting materials

License and Terms: This is a supporting information file under the terms of the Creative Commons Attribution License (http://creativecommons.org/licenses/by/4.0). Please note that the reuse, redistribution and reproduction in particular requires that the authors and source are credited.
The license is subject to the *Beilstein Journal of Organic Chemistry* terms and conditions: (https://www.beilstein-journals.org/bjoc)

Table of contents

1. Supporting Information Figures S1–S6 .... S2
2. Additional docking experiments figures S6
3. Spectra of synthesized glycolipids S8

1. Supporting Information Figures S1–S6

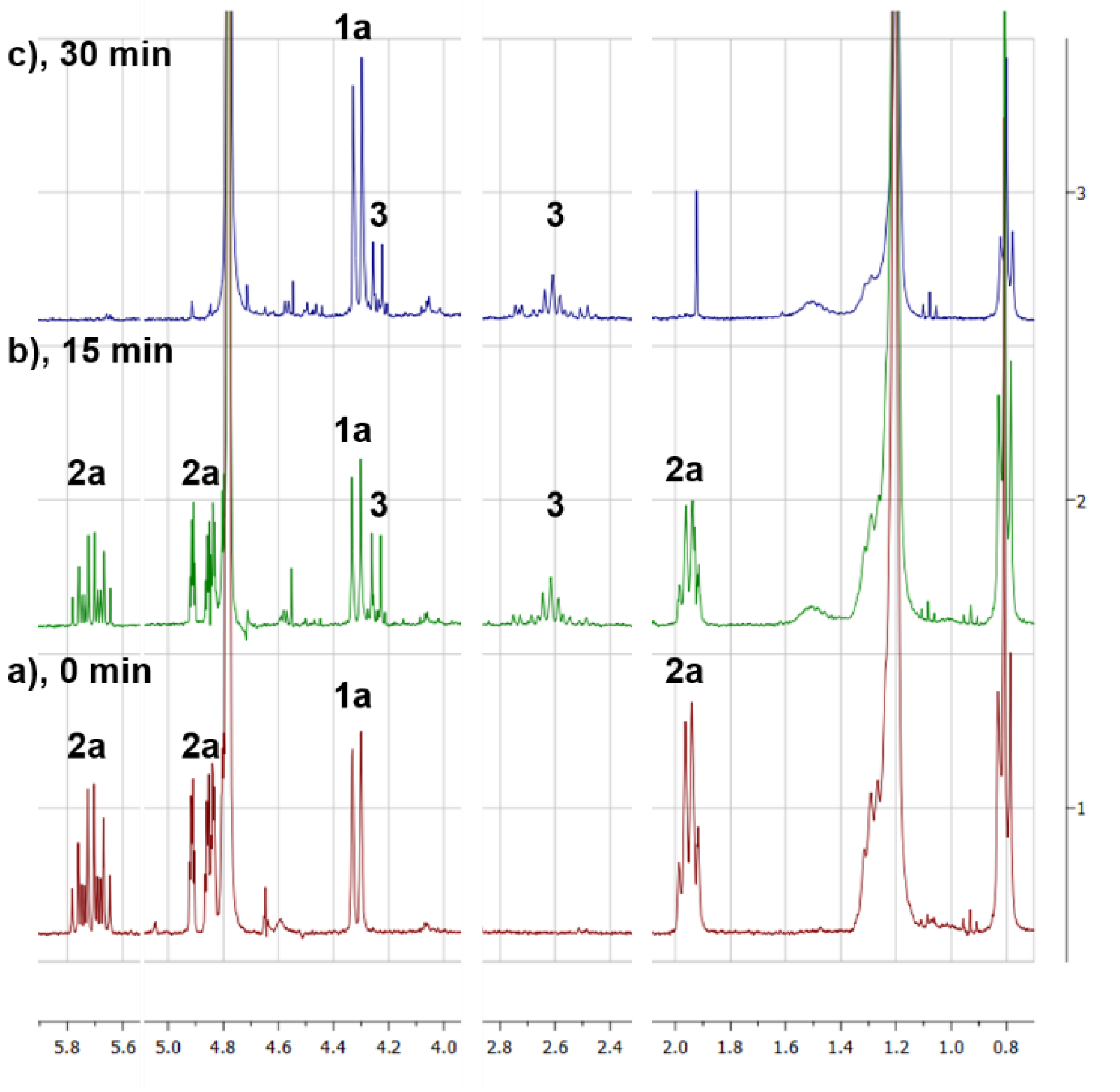

Figure S1. Periodic ^{1}H NMR (300 MHz, CD_3OD) monitoring of the formation of product **3** under TEC conditions.

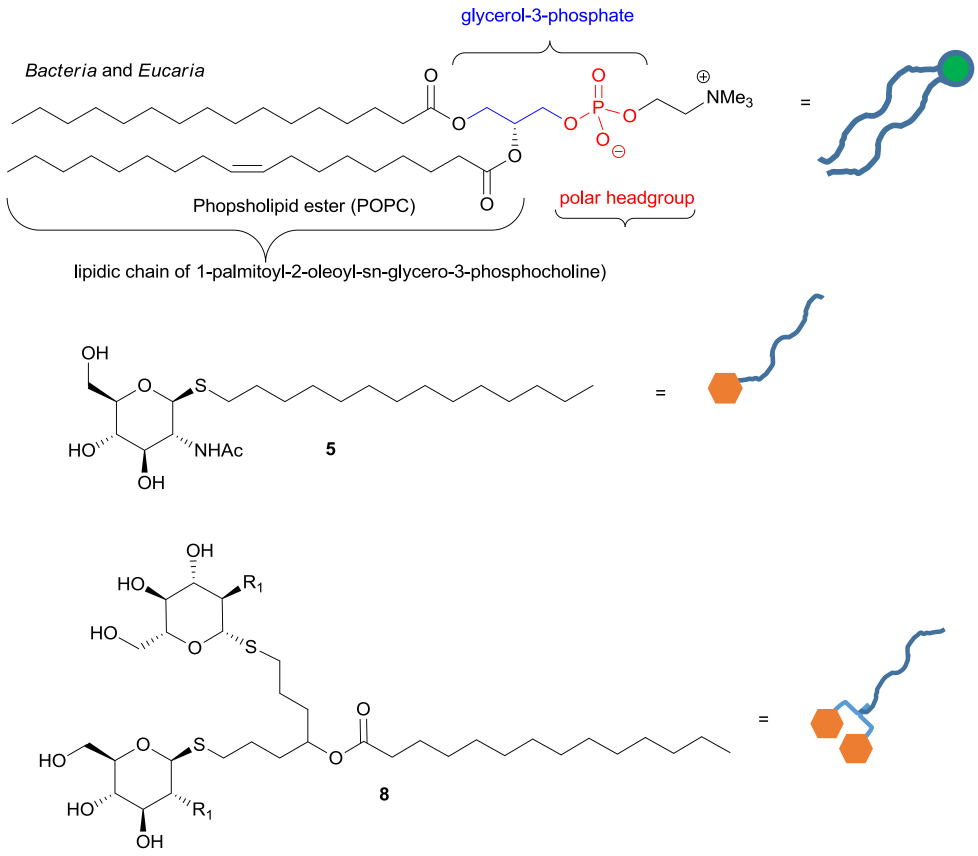

Figure S2. Graphical representation of the amphiphiles used in this research.

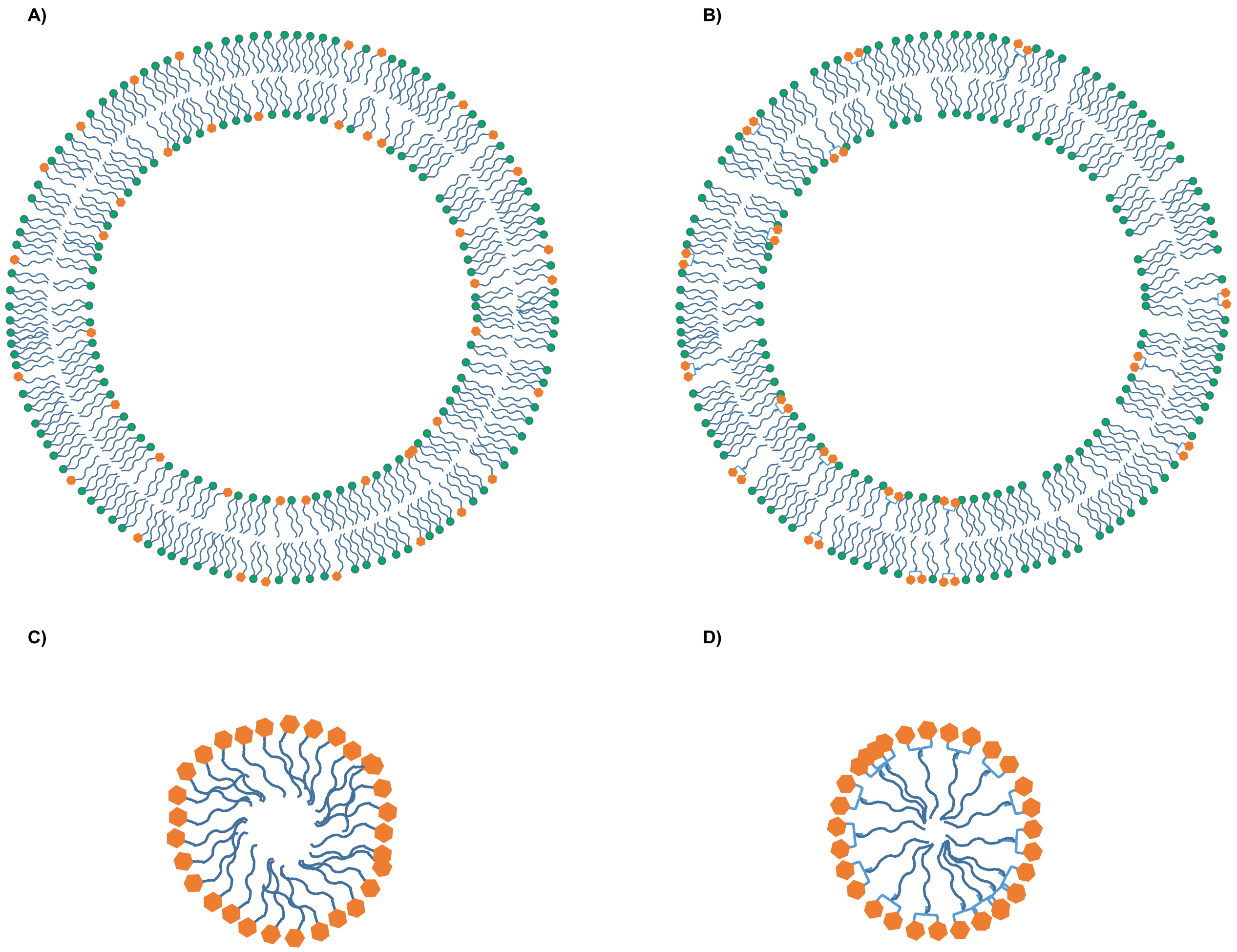

Figure S3. Graphical representation (not in scale) of the giant vesicles (A and B) and of the micelles (C and D) obtained by hydration of a thin film of POPC, **5** and **8** or suspending **5** or **8** in PBS buffer at pH 7.4.

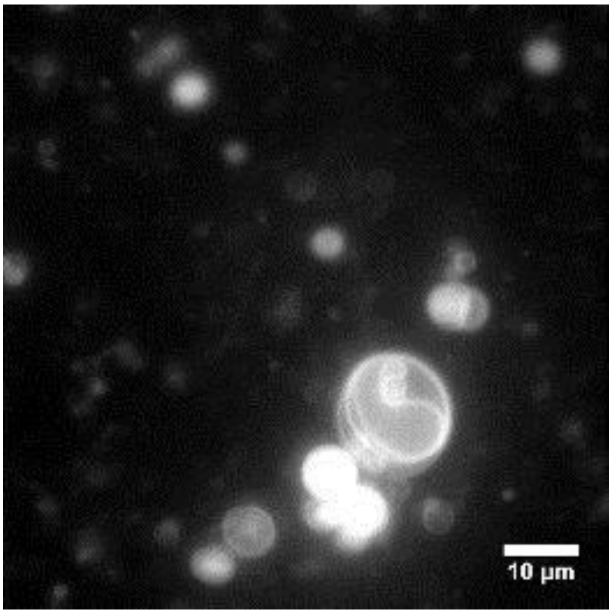

Figure S4. Picture of multilamellar GVs obtained by hydrating pure POPC in PBS buffer at pH 7.4. The sample was stained with Nile Red® (1 mM in DMSO, 1 µL, $\lambda_{[excit]}$ = 561 nm) before the microscopic observation. This structure is very similar to that observed by Holst et al., (Reference 1, page B5) and to that reported in Figure 3 of the present article.

2. Additional docking experiments figures

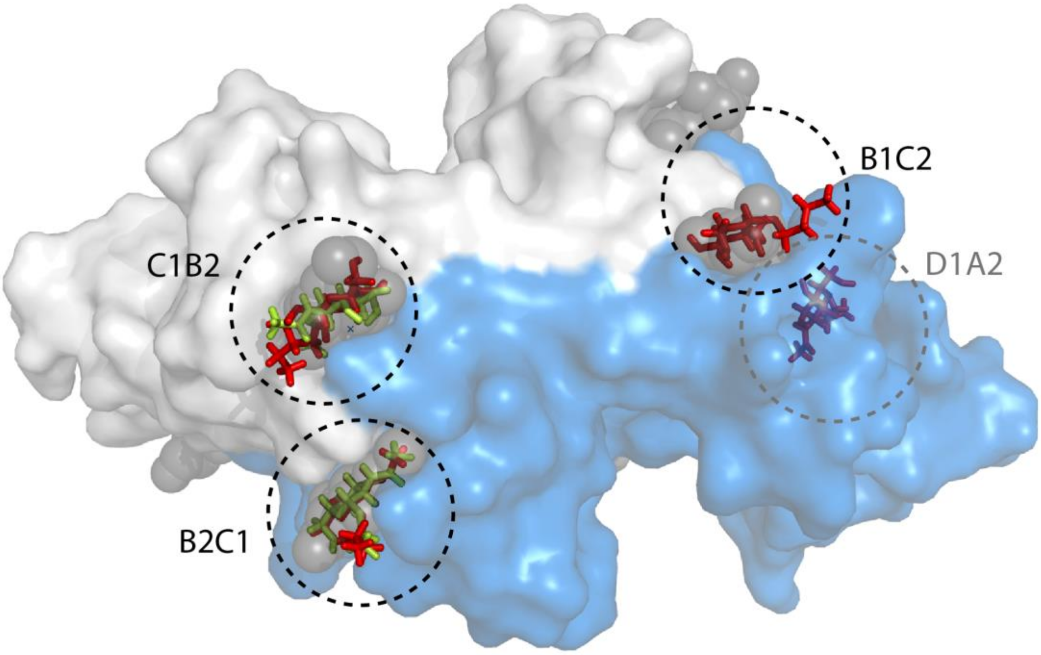

Figure S5: Main poses obtained for docking of **$5C_2$** (green) and **$5C_4$** (red) on 2UVO. GlcNAc are positioned as transparent grey spheres. Ligands are principally recovered on primary binding sites (C1B2, B2C1 and B1C2) and less frequently on the secondary binding site D1A2.

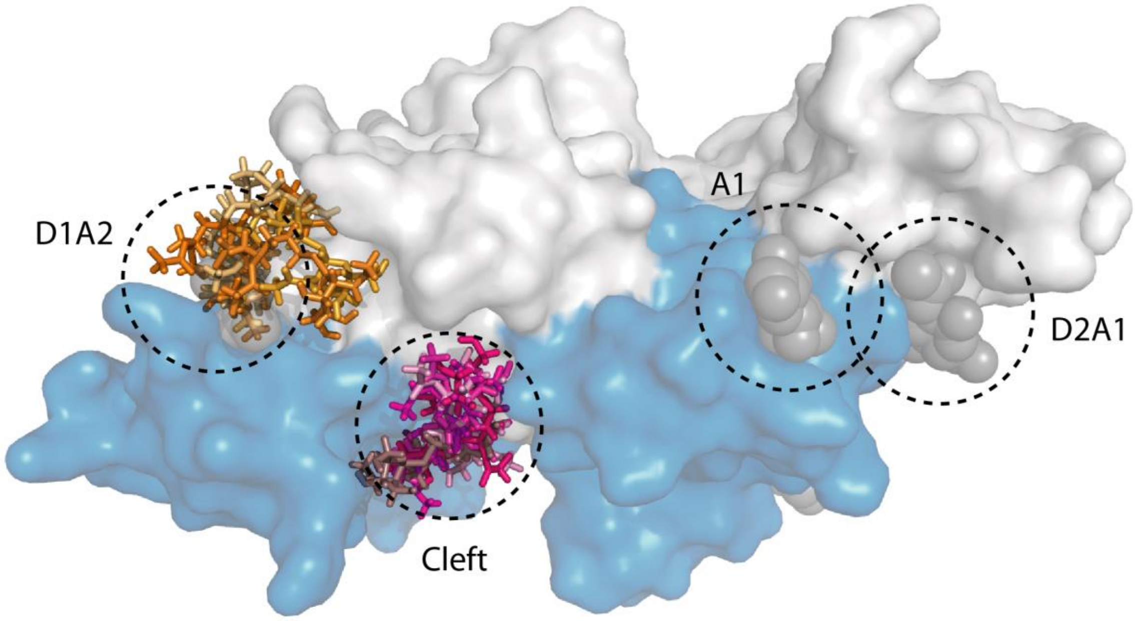

Figure S6: Main poses obtained for docking of **8C$_2$** on 2UVO. GlcNAc are positioned as transparent grey spheres. Positions found on the D1A2 are shown as orange sticks while the position found in the cleft between the two WGA monomers are in pink-purple sticks.

3. Spectra of synthesized glycolipids

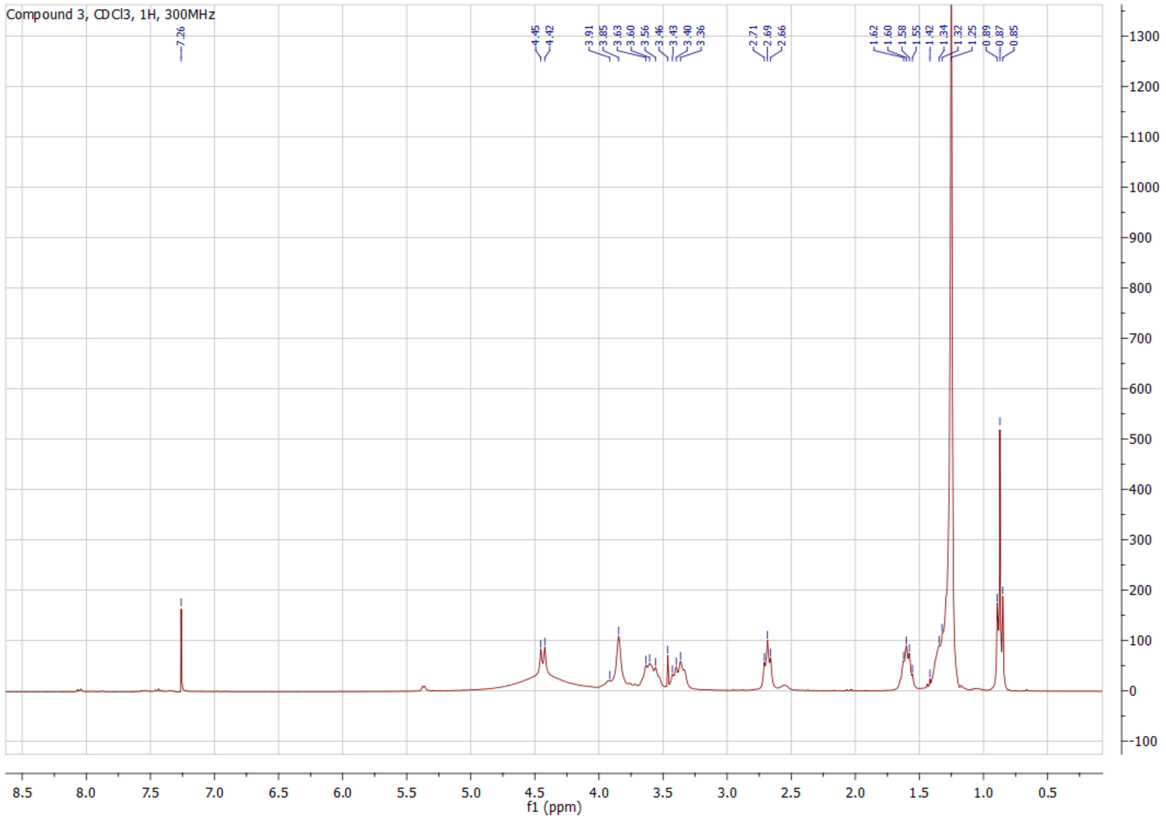

^{1}H NMR (300 MHz, $CDCl_3$) of compound 3

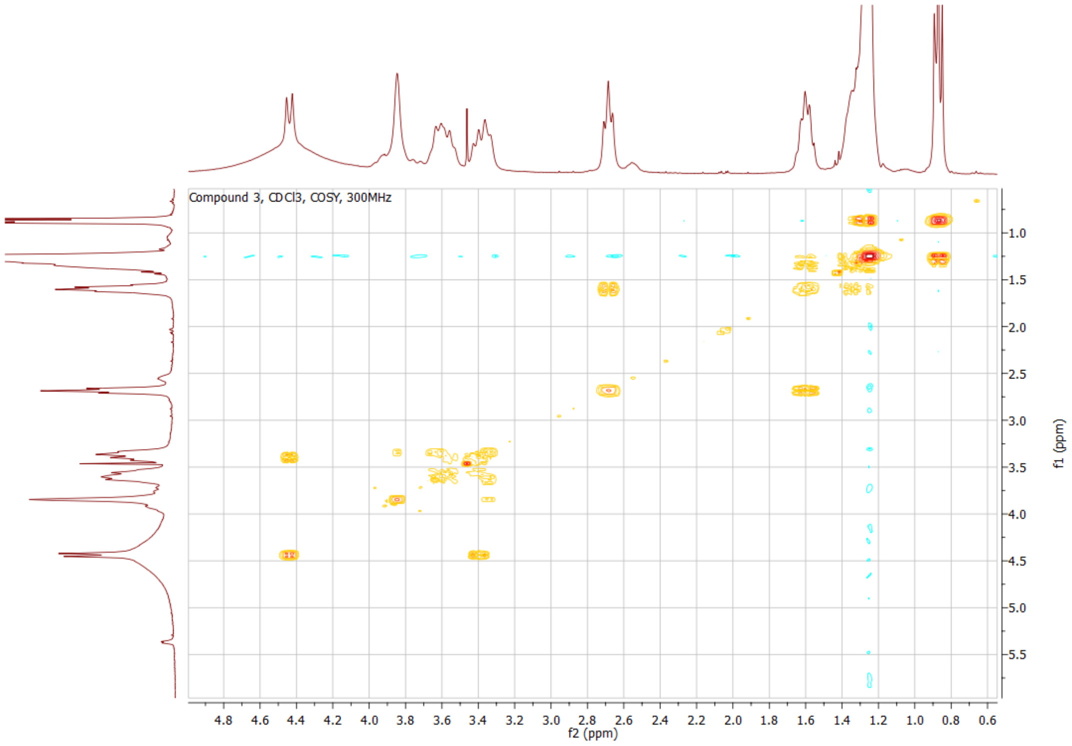

COSY (300 MHz, $CDCl_3$) of compound 3

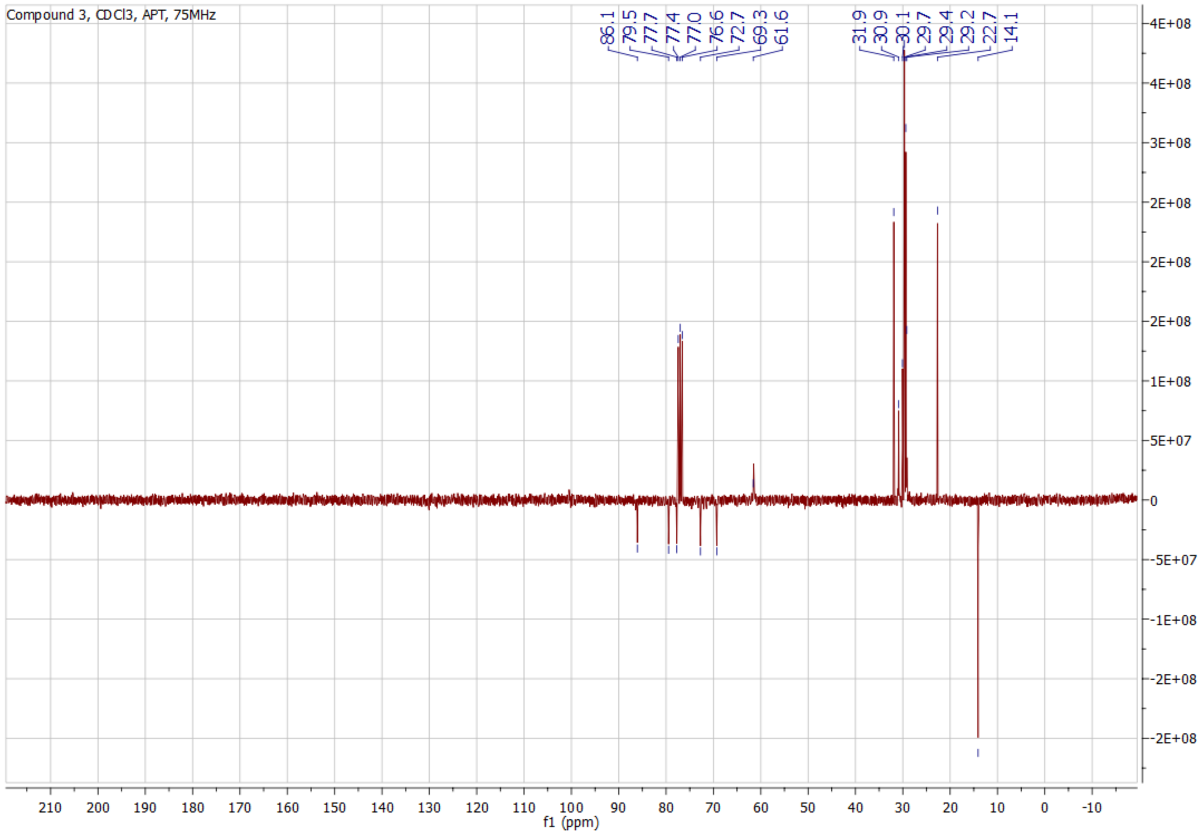

^{13}C APT (75 MHz, $CDCl_3$) of compound 3

GlcNAc-C16 OK

CENTRE COMMUN DE SPECTROMETRIE DE MASSE

Analysis Info

Analysis Name	QTOF170504_04_FADI-A-057-R2.d	Acquisition Date	5/4/2017 4:17:15 PM
Method	2016_03_17_Infusion_50-1000_pos.m	Instrument / Ser#	micrOTOF-Q 228888.10231
Comment			

Acquisition Parameter

Source Type	ESI	Ion Polarity	Positive	Set Nebulizer	0.4 Bar
Focus	Active	Set Capillary	3500 V	Set Dry Heater	200 °C
Scan Begin	50 m/z	Set End Plate Offset	-500 V	Set Dry Gas	4.0 l/min
Scan End	1000 m/z	Set Collision Cell RF	400.0 Vpp	Set Divert Valve	Waste

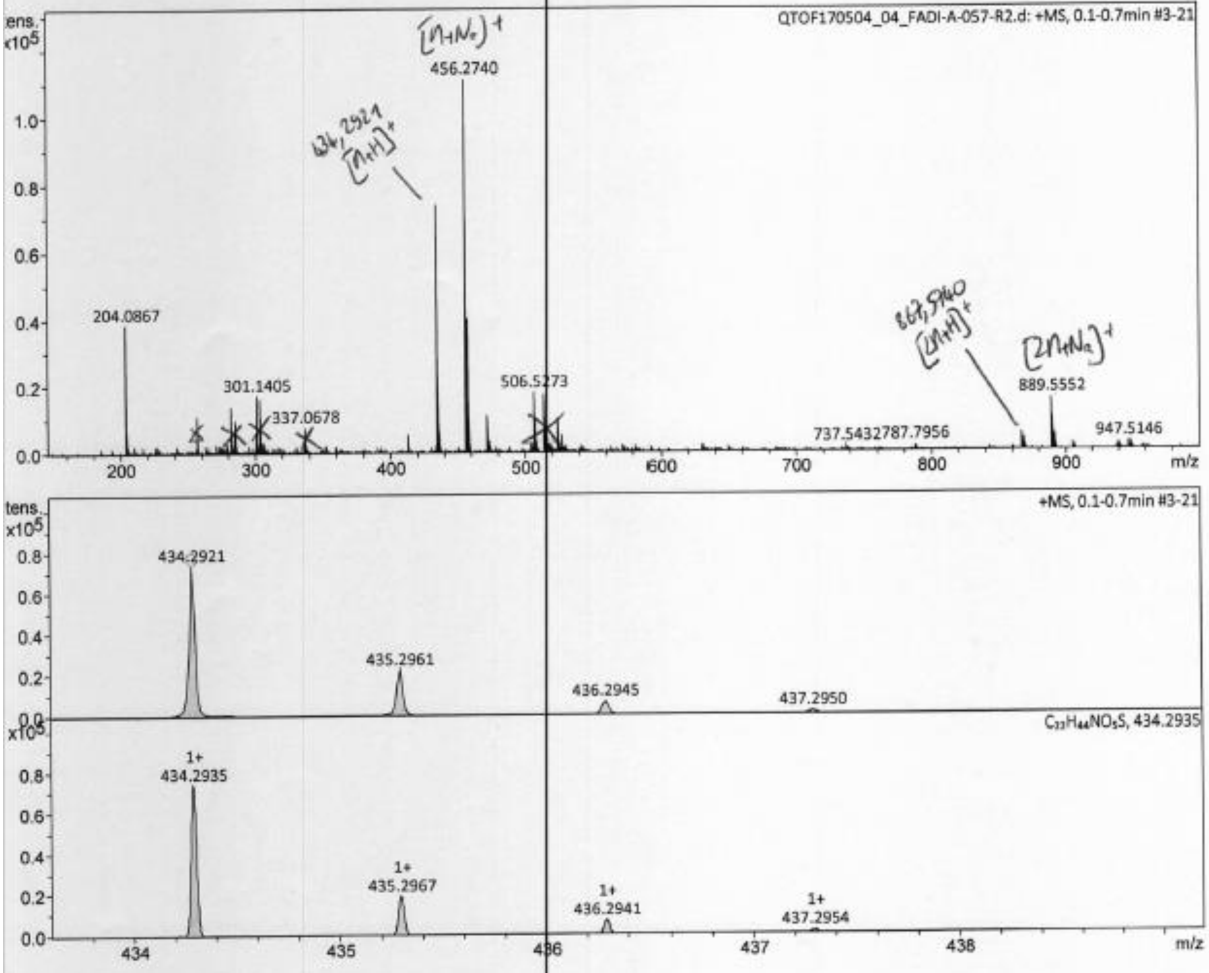

Meas. m/z	Ion Formula	m/z	err [ppm]	mSigma
434.2921	C22H44NO5S	434.2935	3.1	23.3

HRMS of compound 3

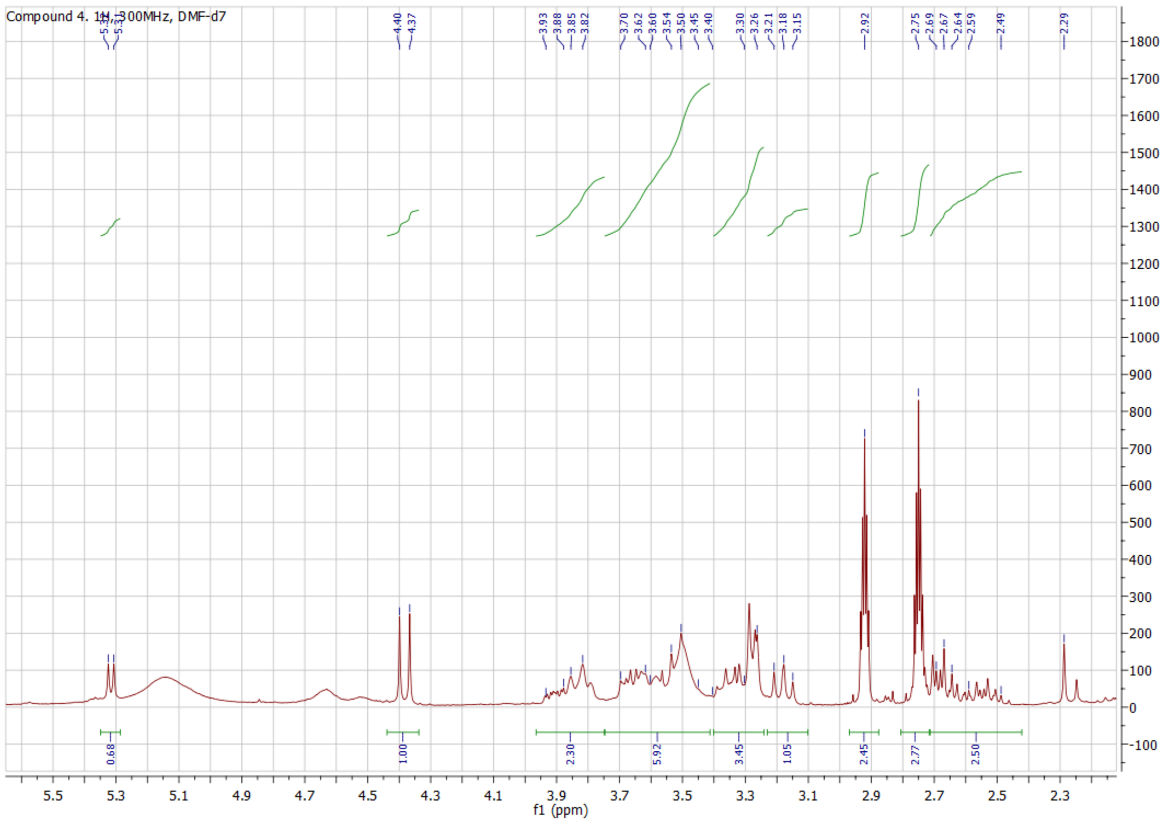

^{1}H NMR (300 MHz, DMF-d_7) of compound 4

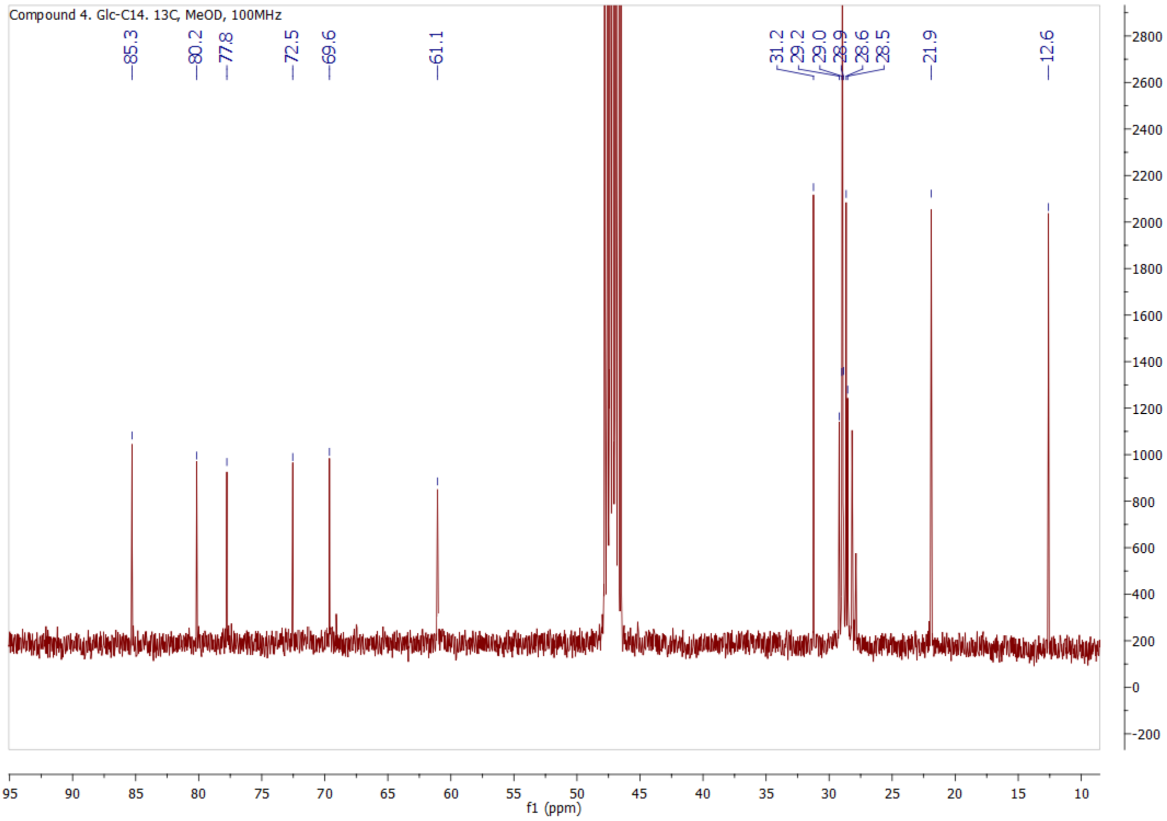

^{13}C NMR (75 MHz, CD_3OD) of compound 4

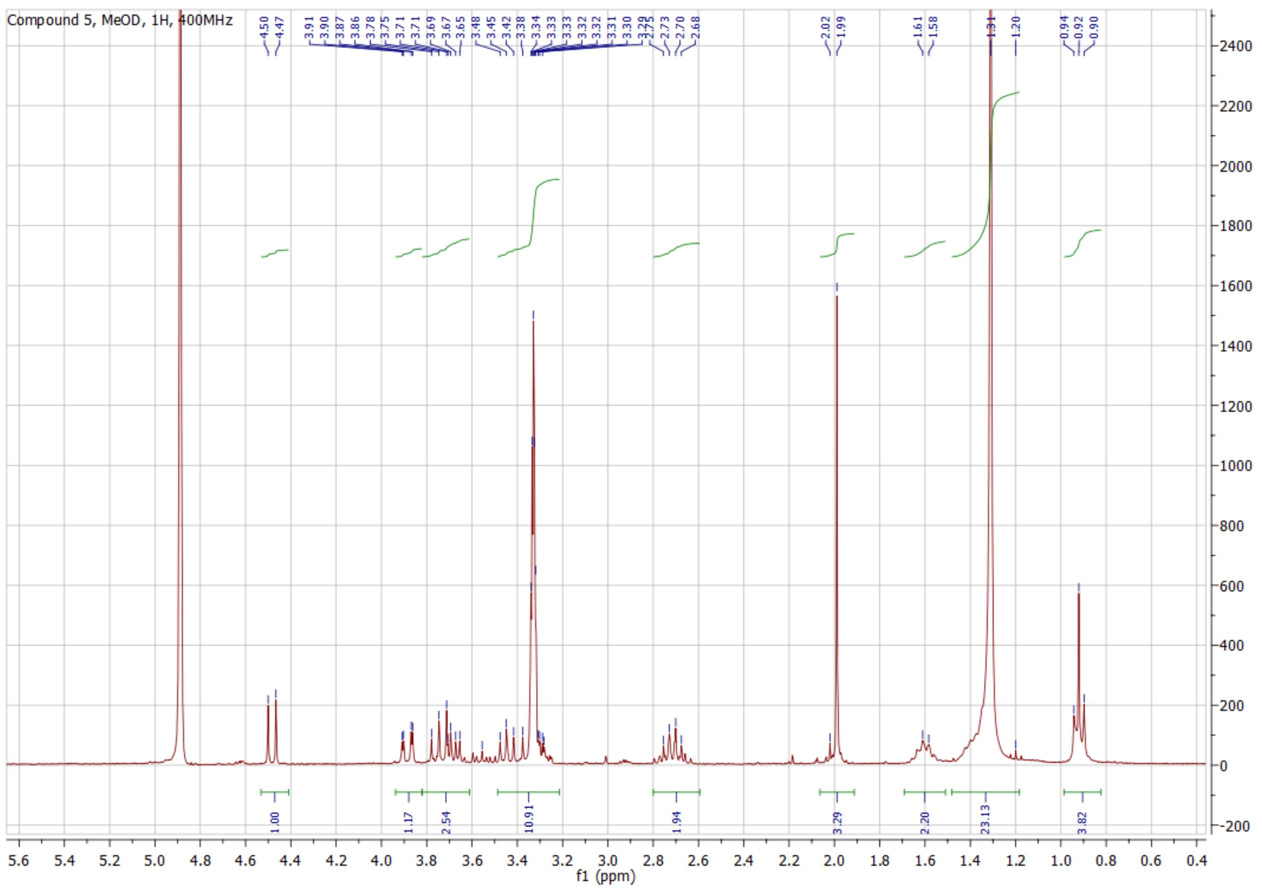

^{1}H NMR (400 MHz, CD_3OD) of compound 5

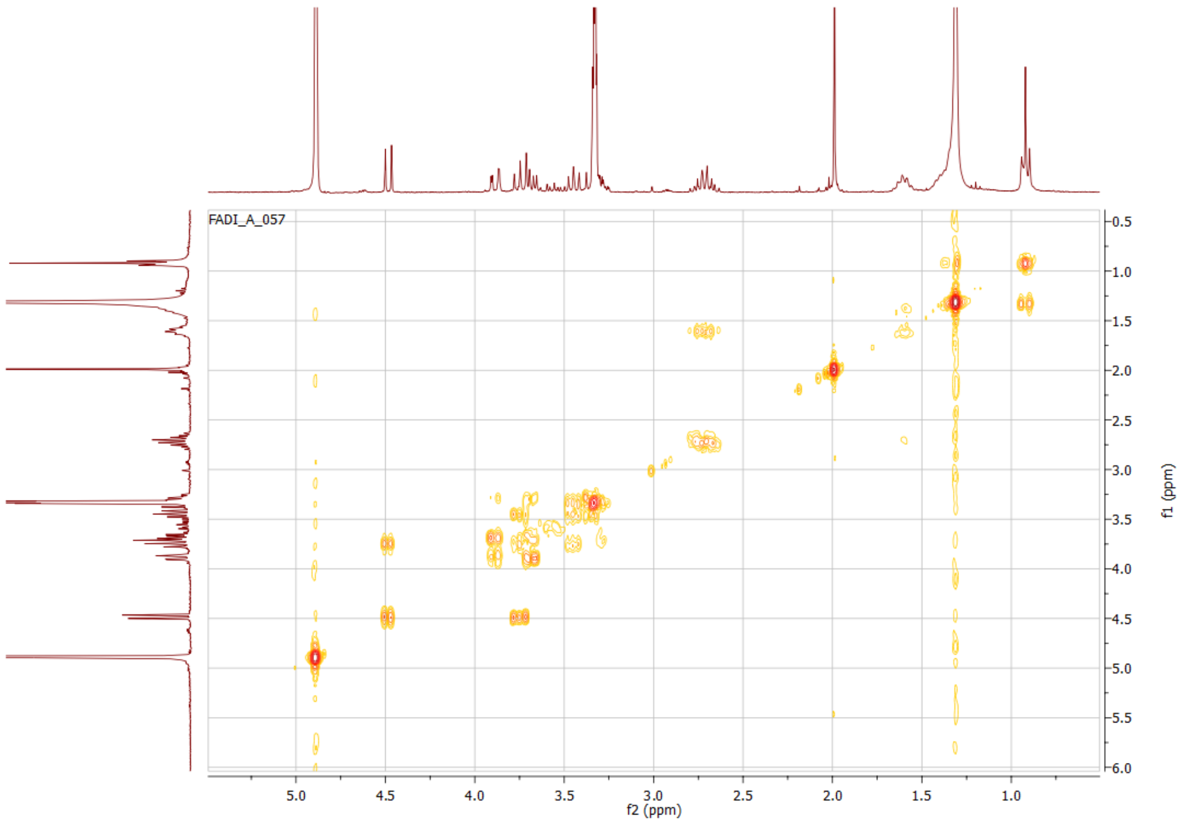

COSY (400 MHz, CD_3OD) of compound 5

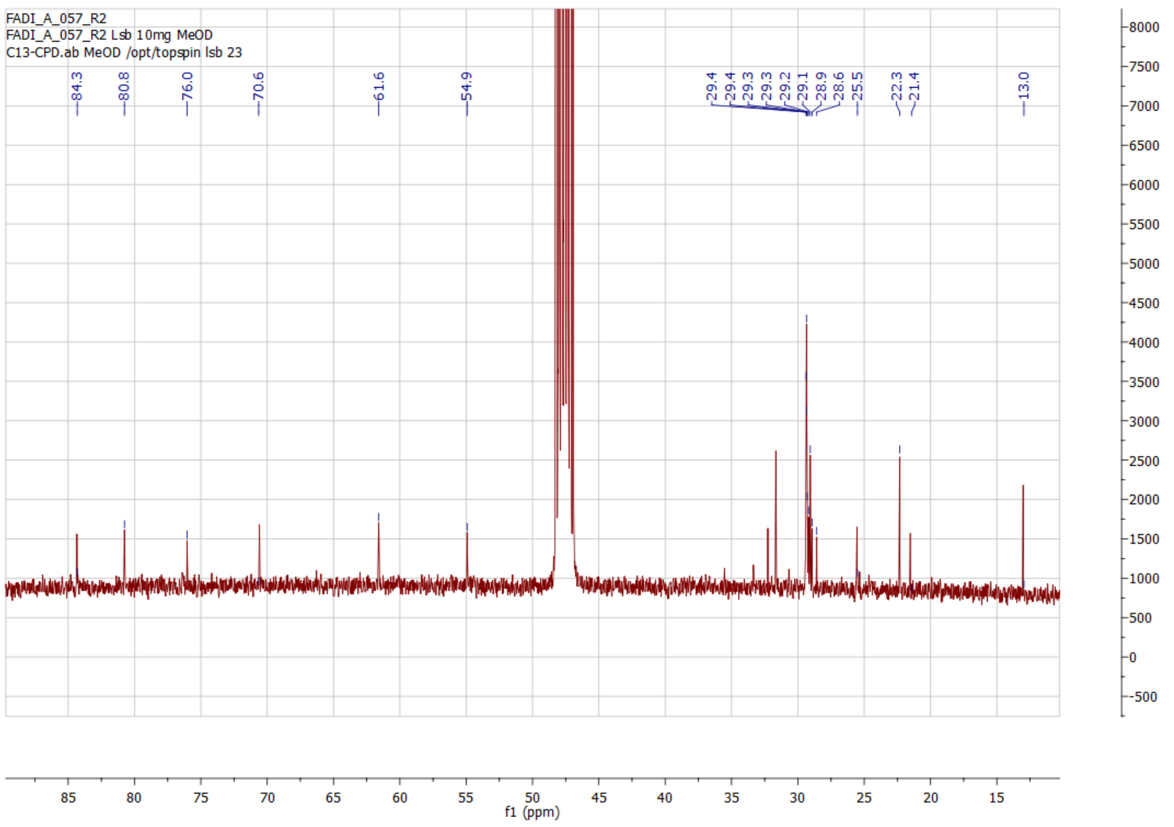

^{13}C NMR (100 MHz, CD_3OD) of compound 5

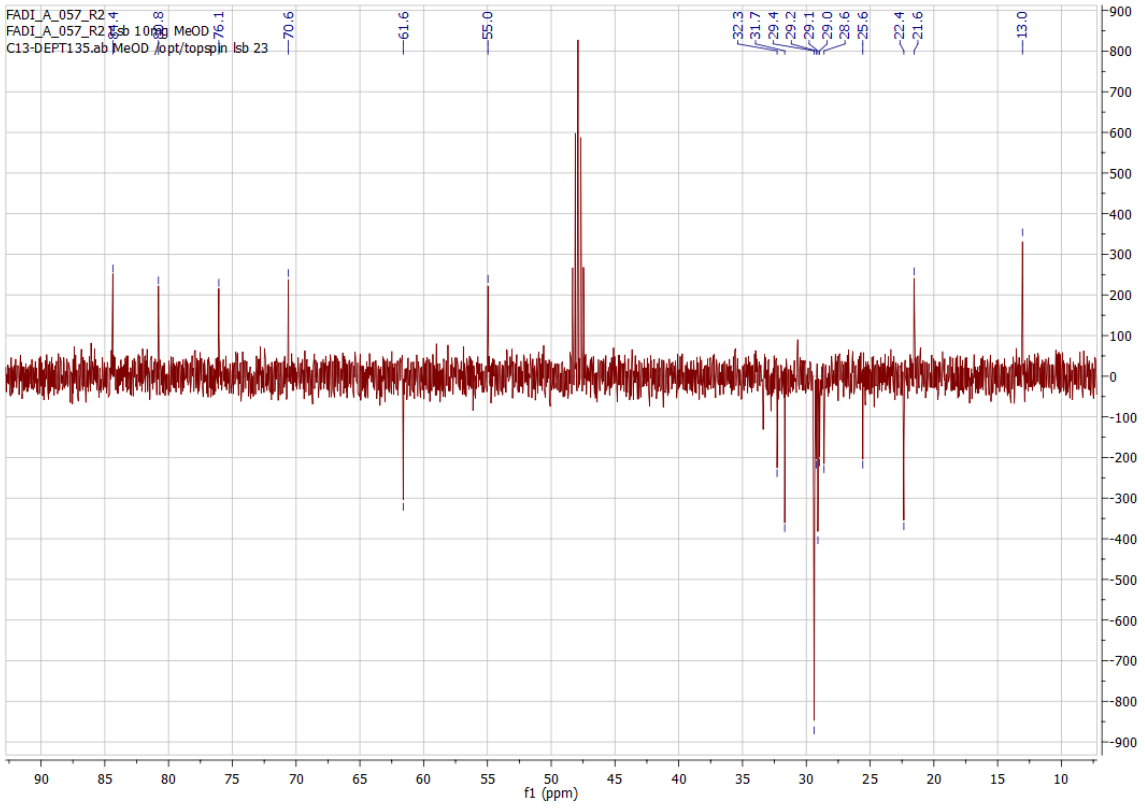

DEPT (100 MHz, CD_3OD) of compound 5

GlcNAc-C₁₄ OK

CENTRE COMMUN DE SPECTROMETRIE DE MASSE

Analysis Info

Analysis Name	QTOF170504_04_FADI-A-057-R2.d	Acquisition Date	5/4/2017 4:17:15 PM
Method	2016_03_17_Infusion_50-1000_pos.m	Instrument / Ser#	micrOTOF-Q 228888.10231
Comment			

Acquisition Parameter

Source Type	ESI	Ion Polarity	Positive	Set Nebulizer	0.4 Bar
Focus	Active	Set Capillary	3500 V	Set Dry Heater	200 °C
Scan Begin	50 m/z	Set End Plate Offset	-500 V	Set Dry Gas	4.0 l/min
Scan End	1000 m/z	Set Collision Cell RF	400.0 Vpp	Set Divert Valve	Waste

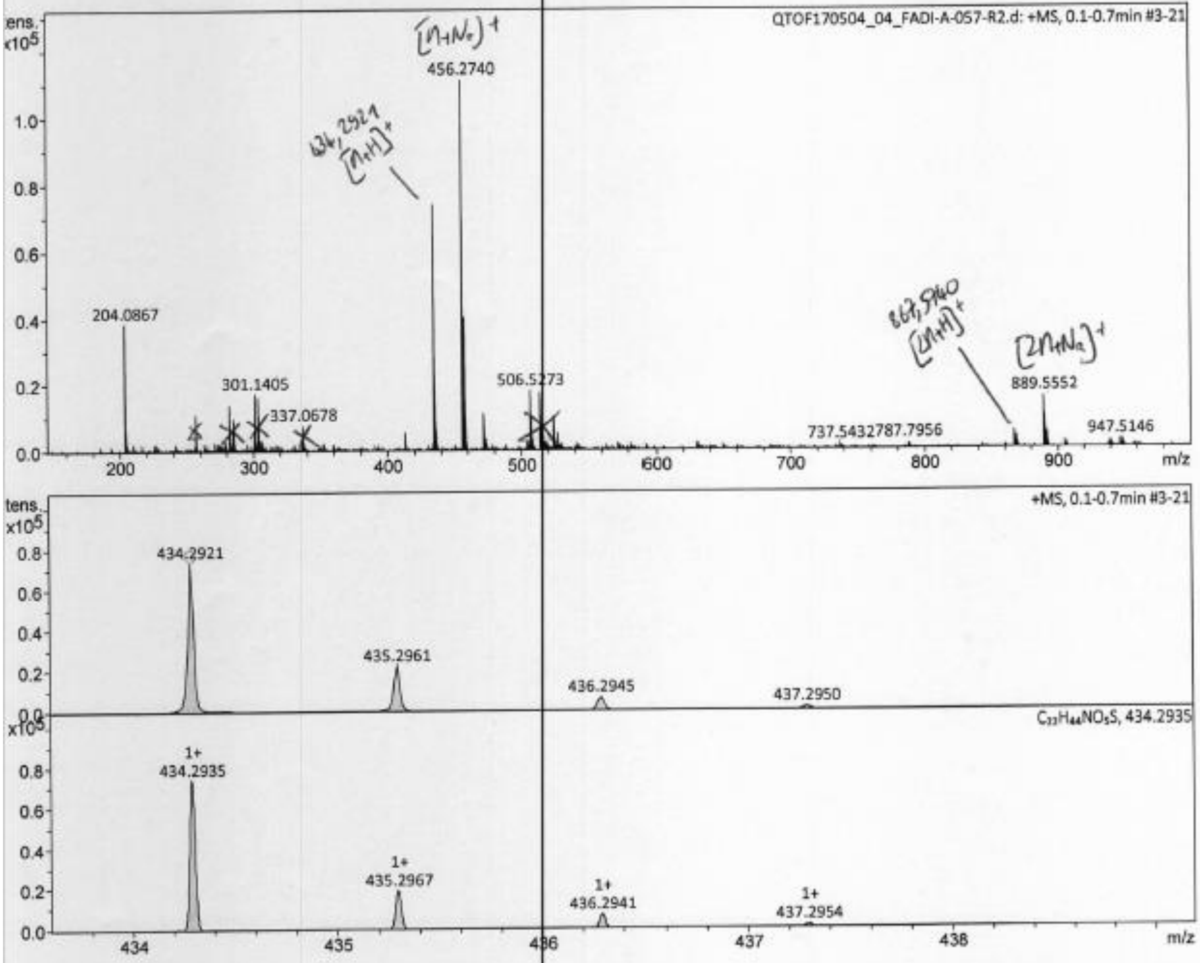

Meas. m/z	Ion Formula	m/z	err [ppm]	mSigma
434.2921	C22H44NO5S	434.2935	3.1	23.3

HRMS of compound 5

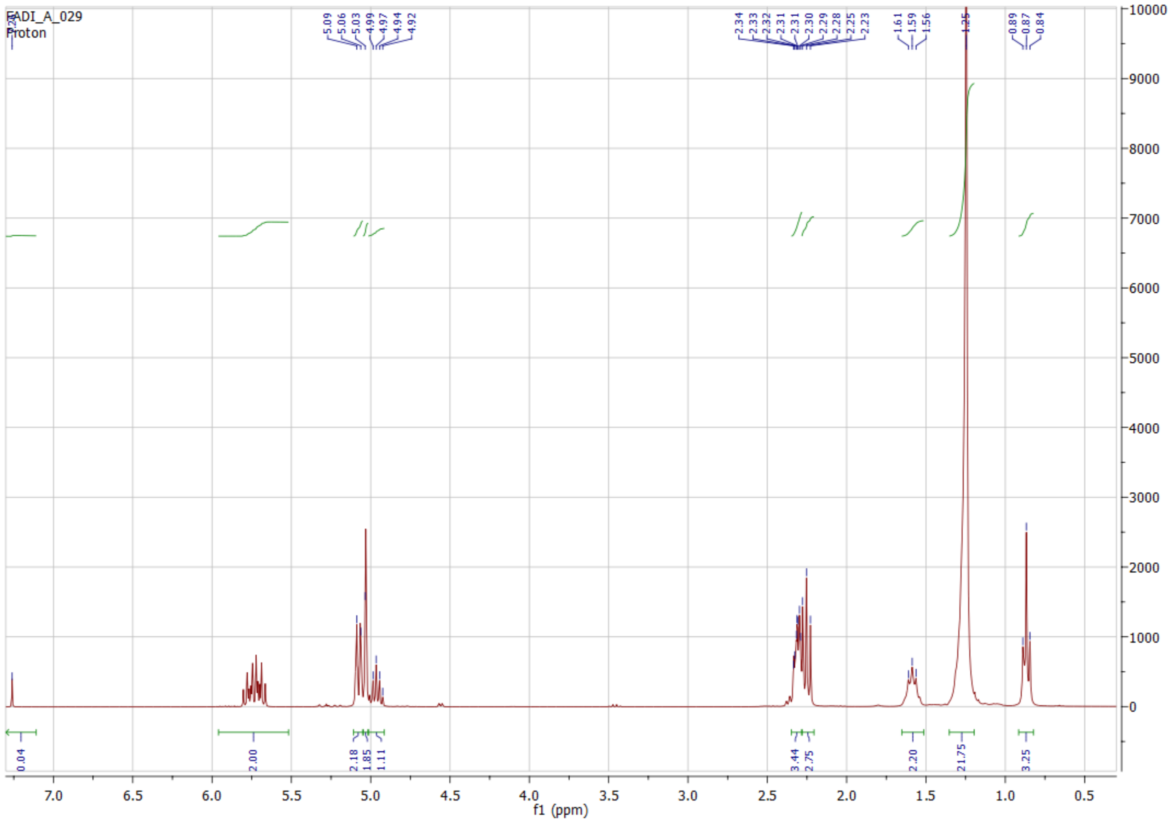

^{1}H NMR (300 MHz, $CDCl_3$) of compound 6

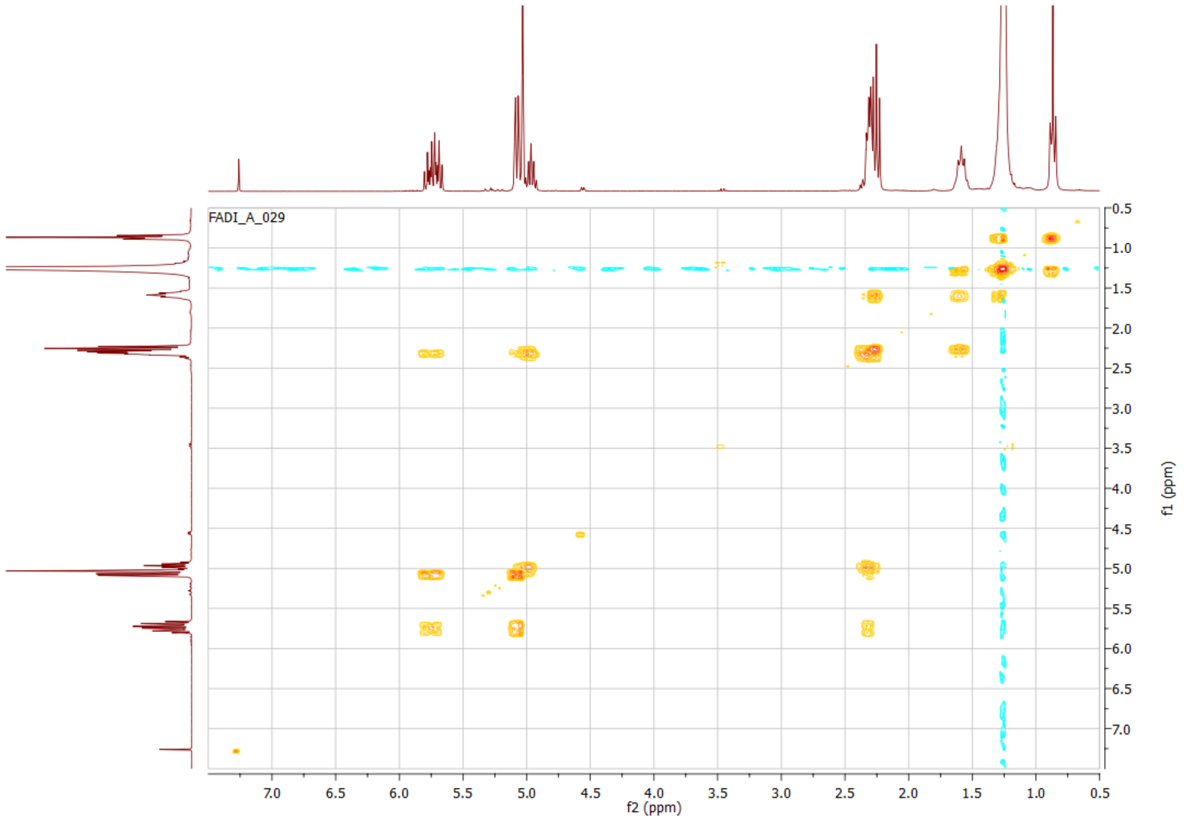

COSY (300 MHz, $CDCl_3$) of compound 6

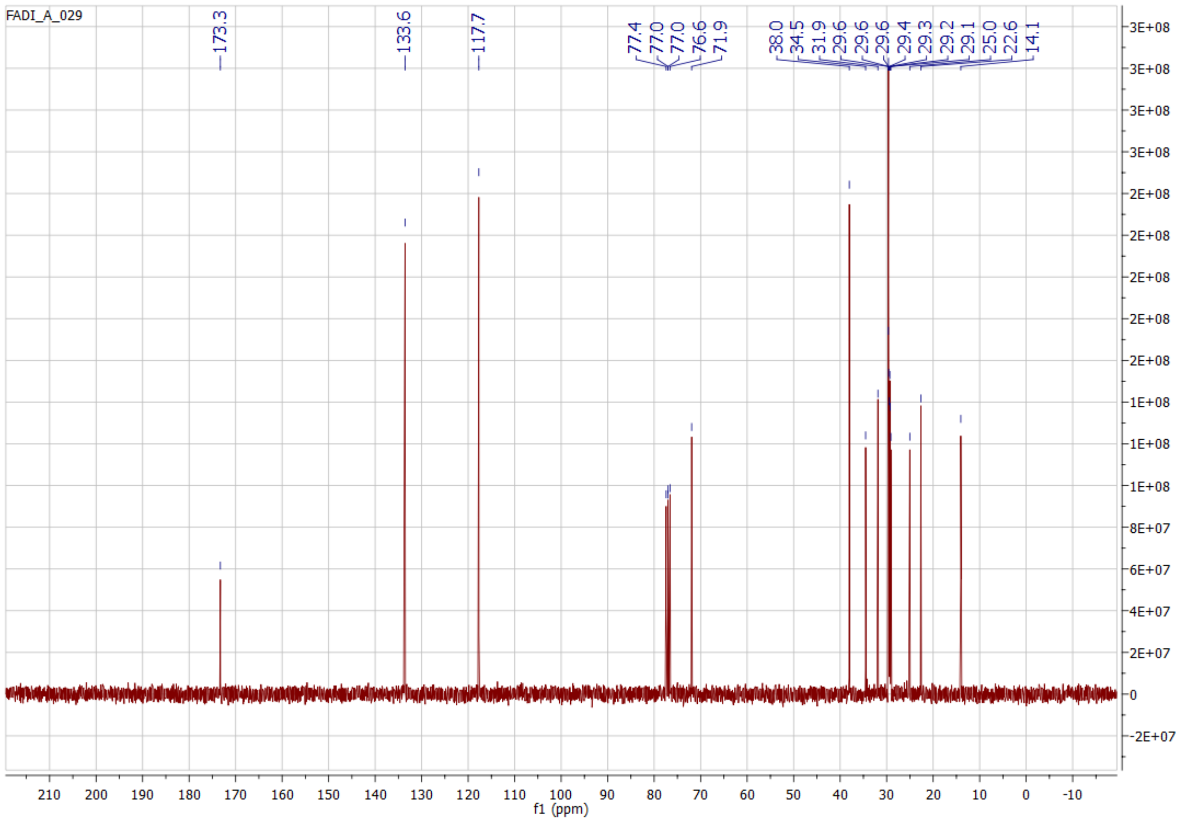

^{13}C NMR (75 MHz, $CDCl_3$) of compound 6

calc. $C_{21}H_{38}O_2$ 322.525

CENTRE COMMUN DE SPECTROMETRIE DE MASSE

Analysis Info

Analysis Name	QTOF160520_03_FAD1-A-029.d		
Method	2016_03_17_Infusion_50-1000_pos.m	Acquisition Date	5/20/2016 3:17:17 PM
Comment		Instrument / Ser#	micrOTOF-Q 228888.10231

Acquisition Parameter

Source Type	ESI	Ion Polarity	Positive	Set Nebulizer	0.4 Bar
Focus	Active	Set Capillary	3500 V	Set Dry Heater	200 °C
Scan Begin	50 m/z	Set End Plate Offset	-500 V	Set Dry Gas	4.0 l/min
Scan End	1000 m/z	Set Collision Cell RF	400.0 Vpp	Set Divert Valve	Waste

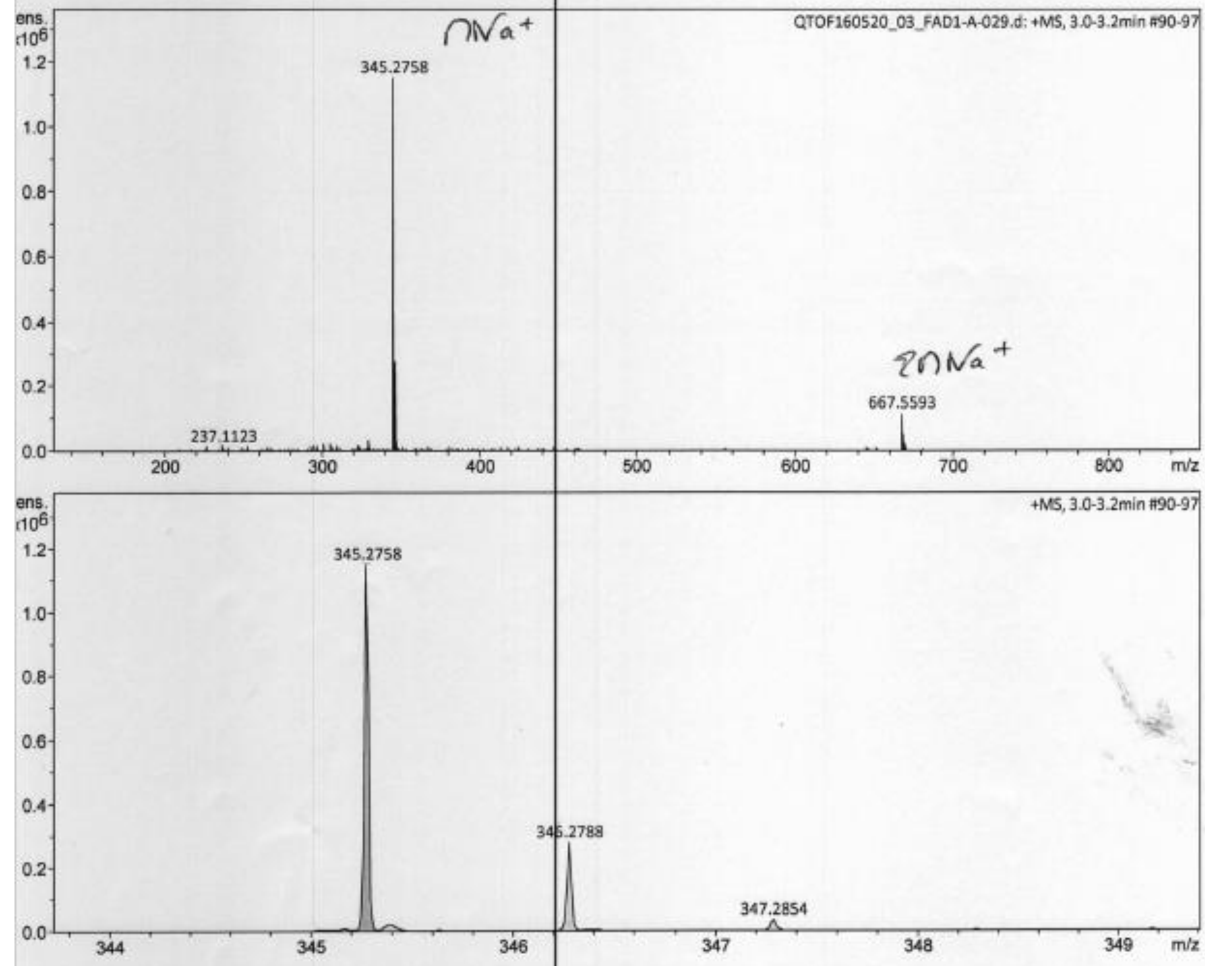

Meas. m/z	Ion Formula	m/z	err [ppm]	mSigma
345.2758	C21H38NaO2	345.2764	1.9	5.7

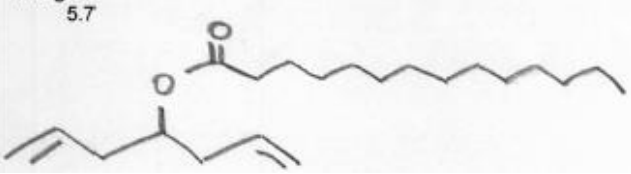

HRMS of compound 6

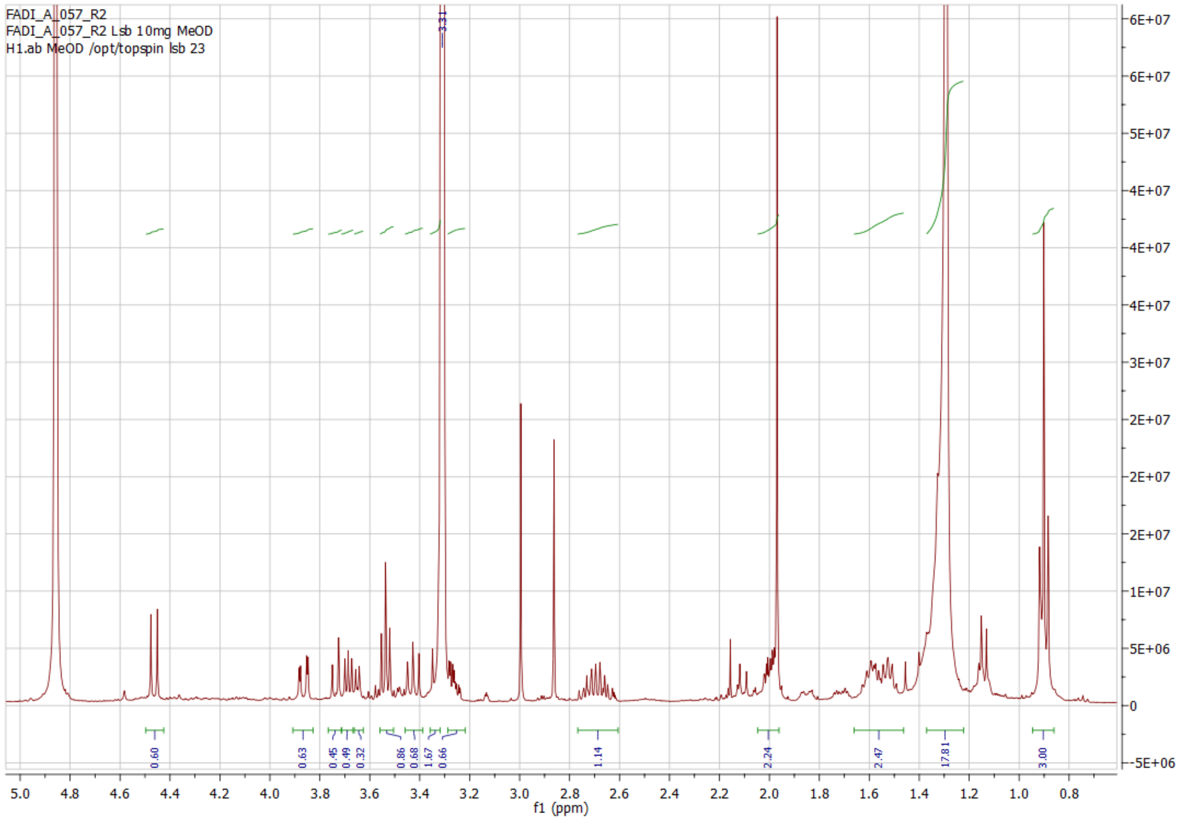

^{1}H NMR (400 MHz, CD_3OD) of compound 7

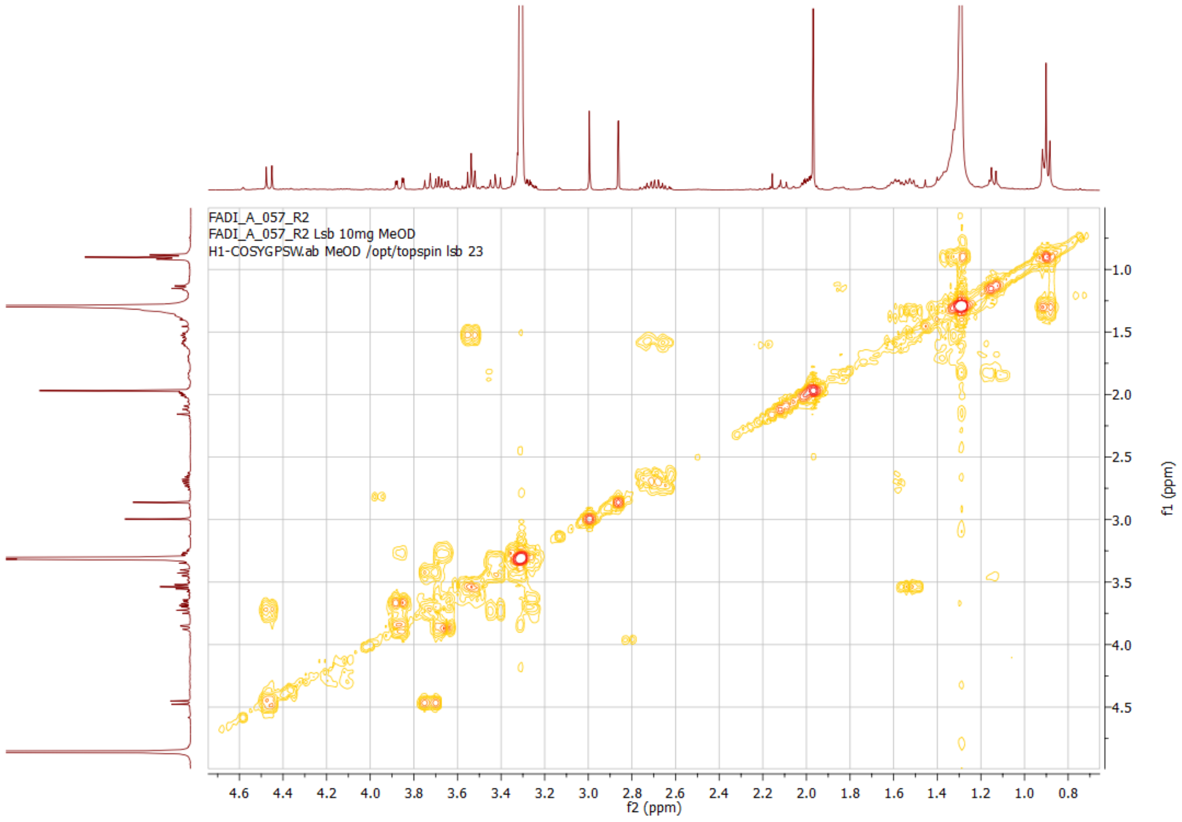

COSY (400 MHz, CD_3OD) of compound 7

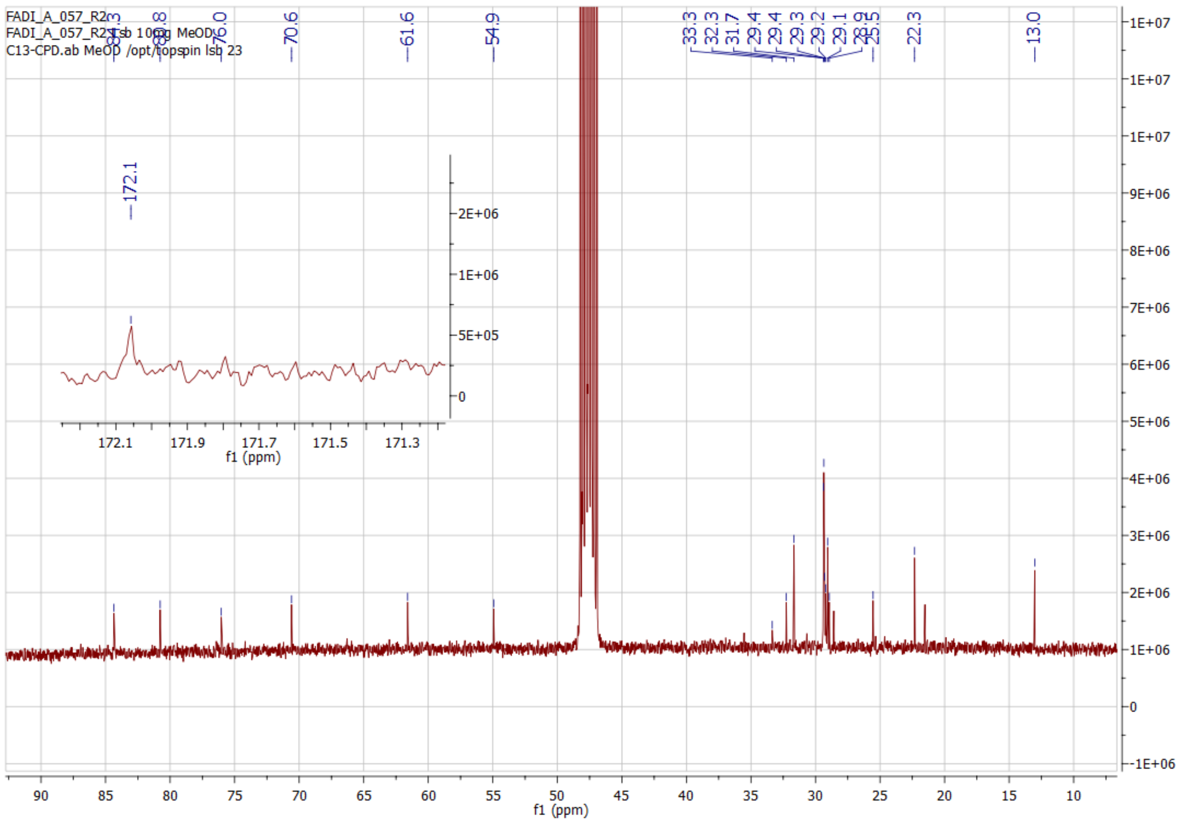

^{13}C NMR (100 MHz, $CDCl_3$) of compound 7

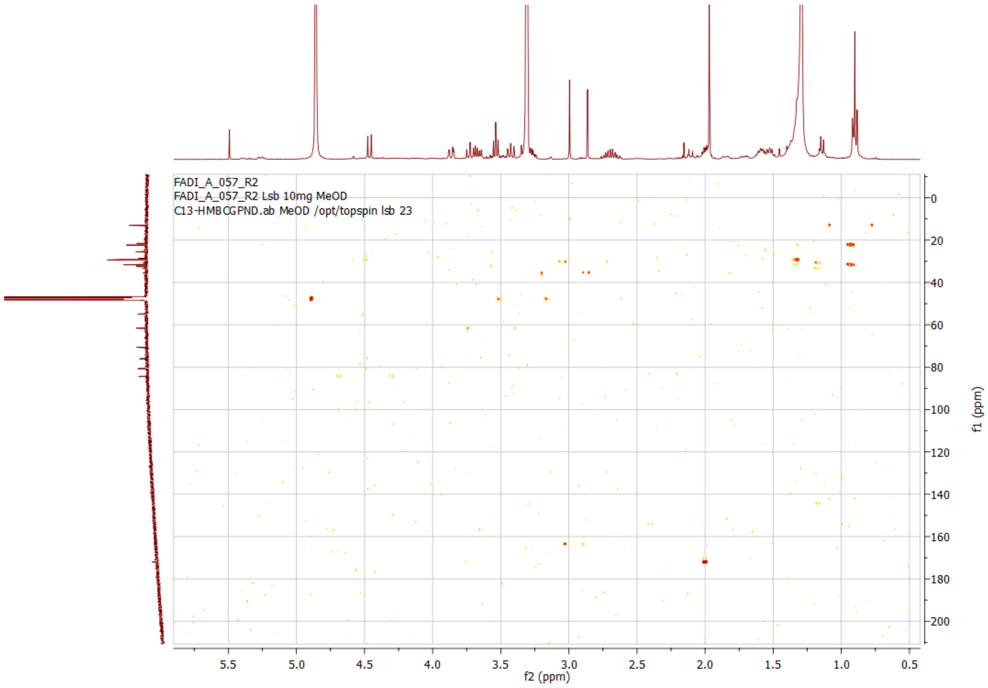

HMBC ($CDCl_3$) of compound 7

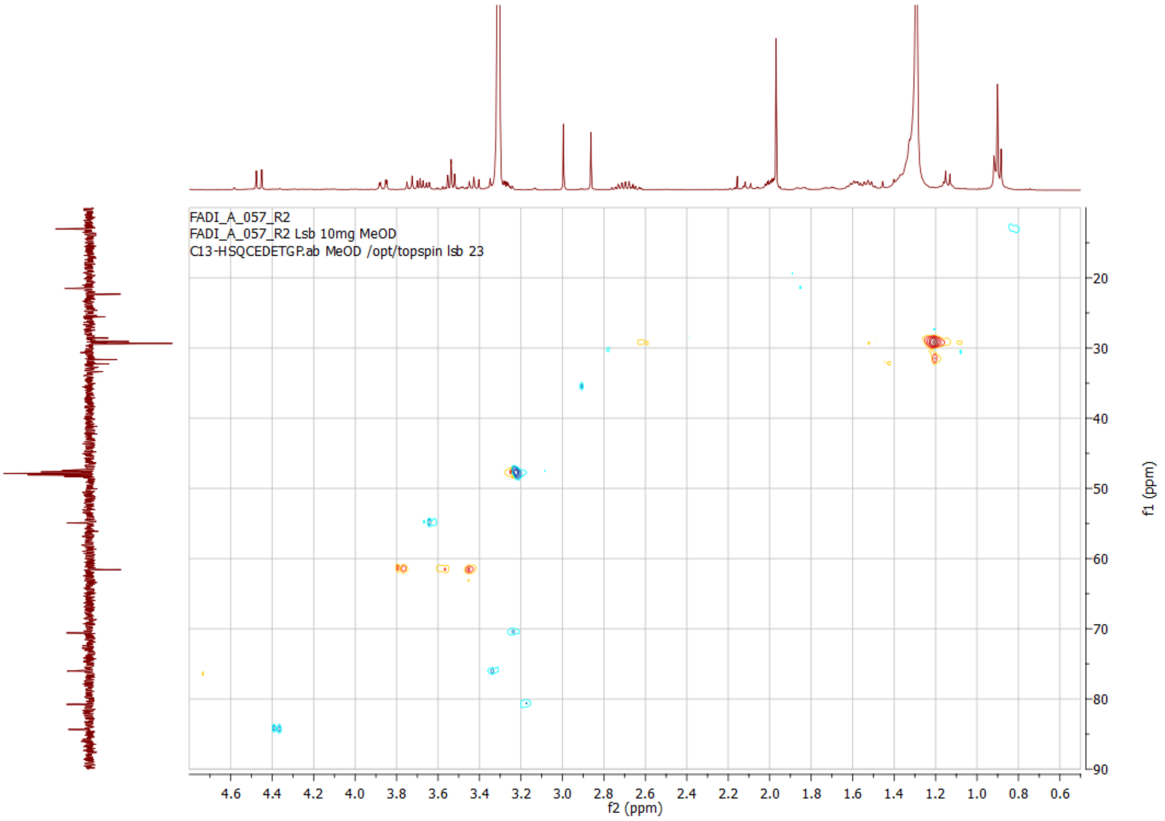

HSQC ($CDCl_3$) of compound 7

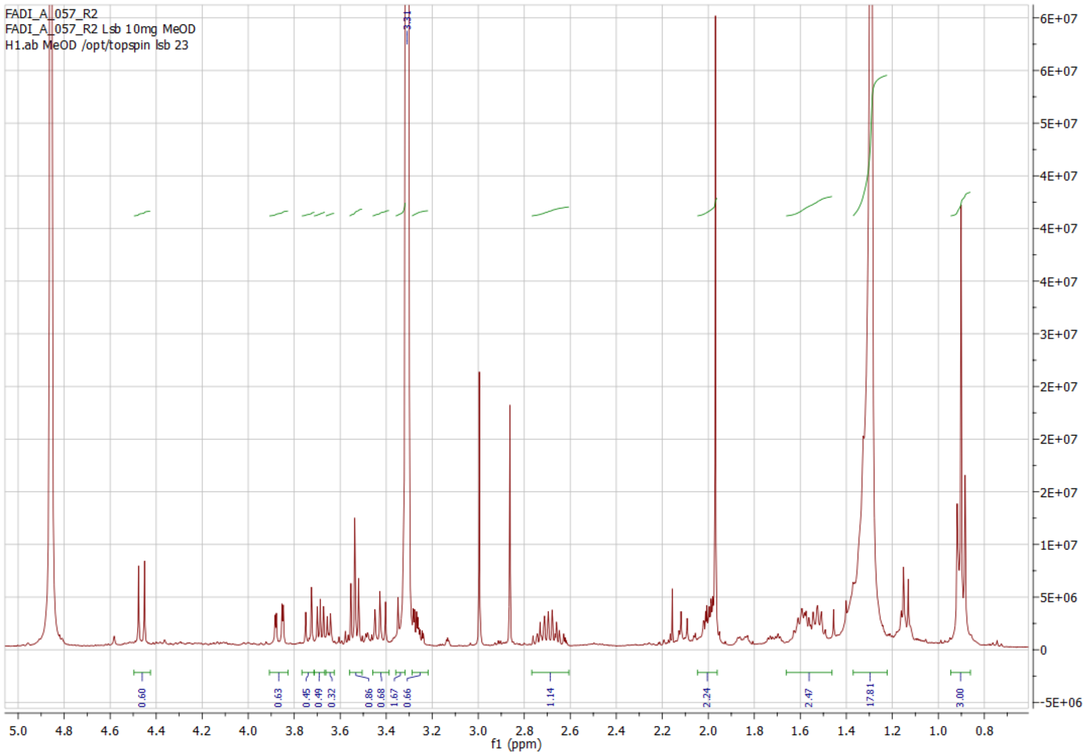

^{1}H NMR (400 MHz, CD_3OD) of compound 8

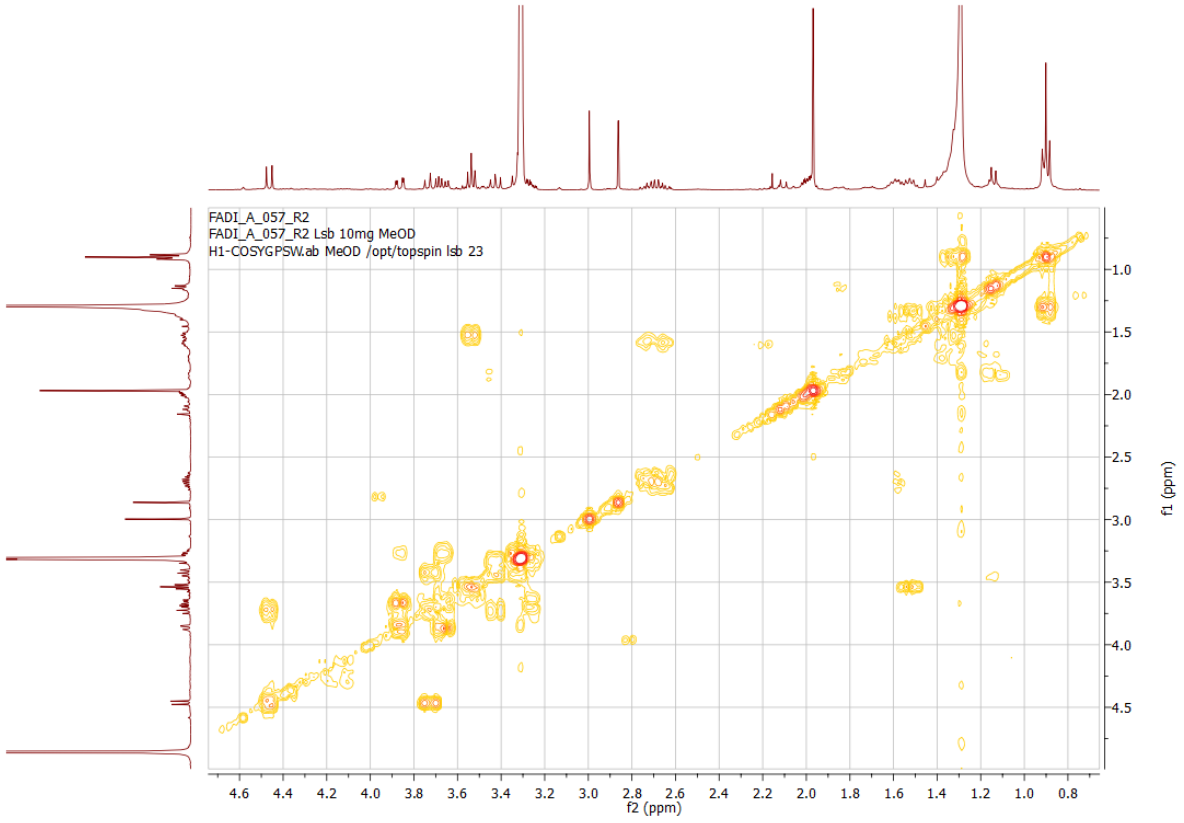

COSY (400 MHz, CD_3OD) of compound 8

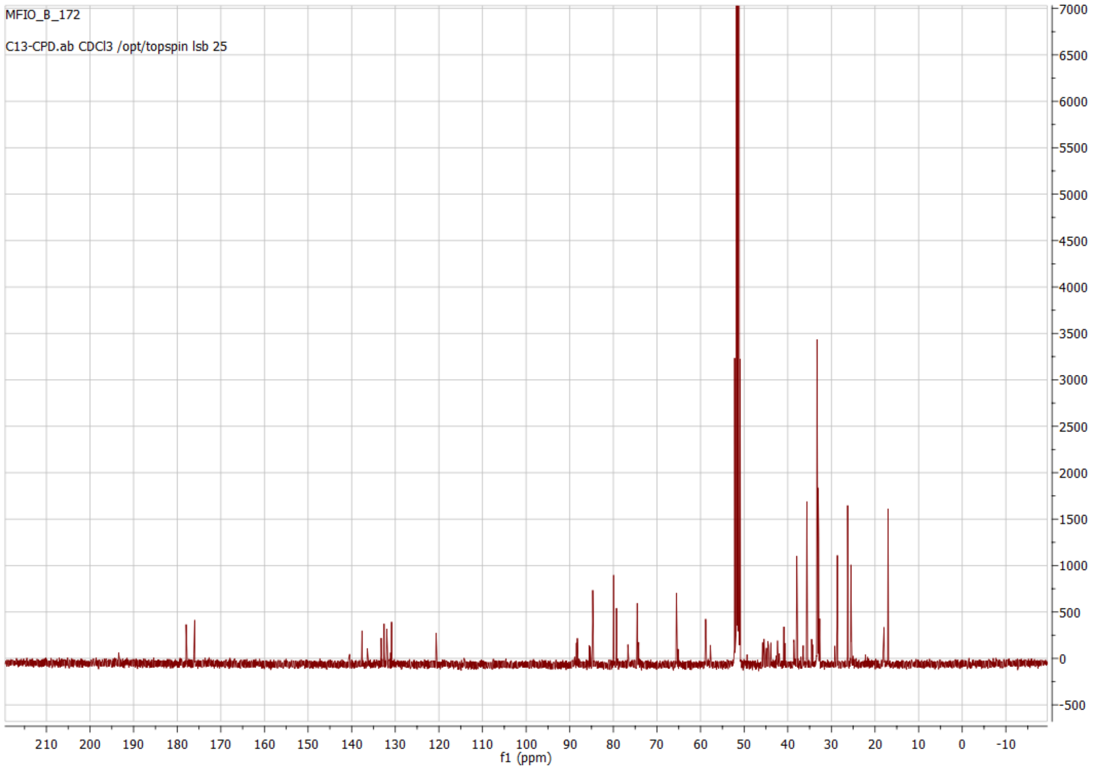

^{13}C NMR (100 MHz, $CDCl_3$) of compound 8

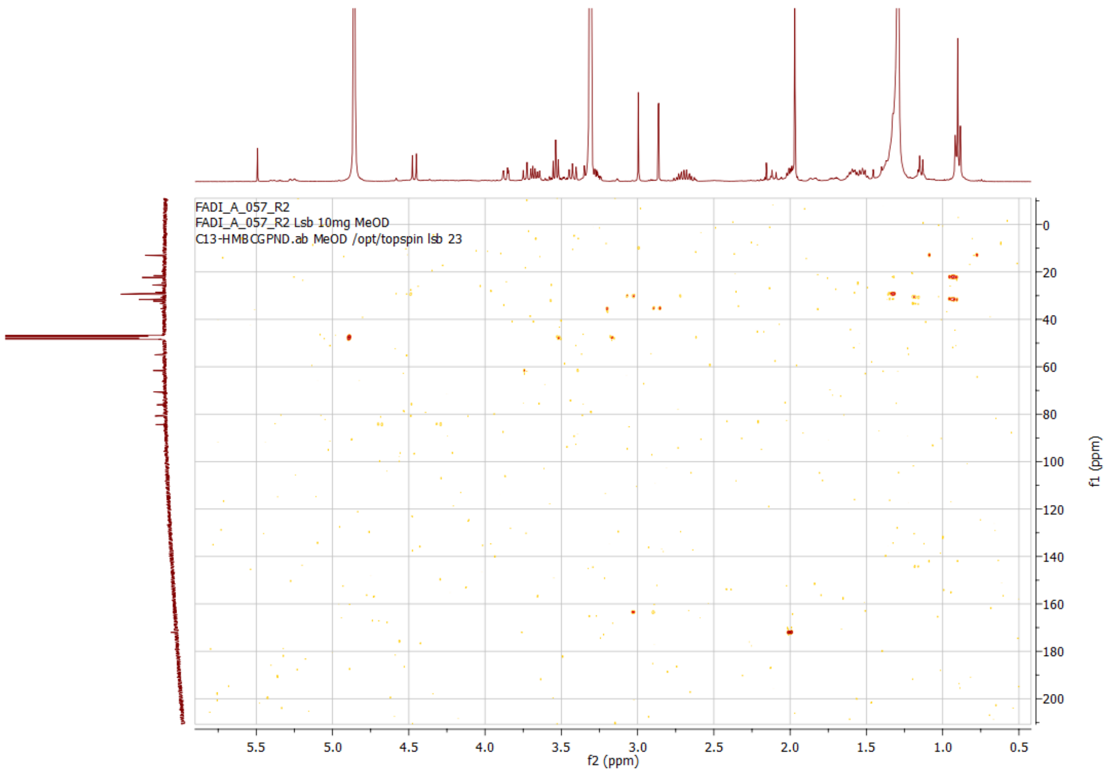

HMBC (CD_3OD) of compound 8

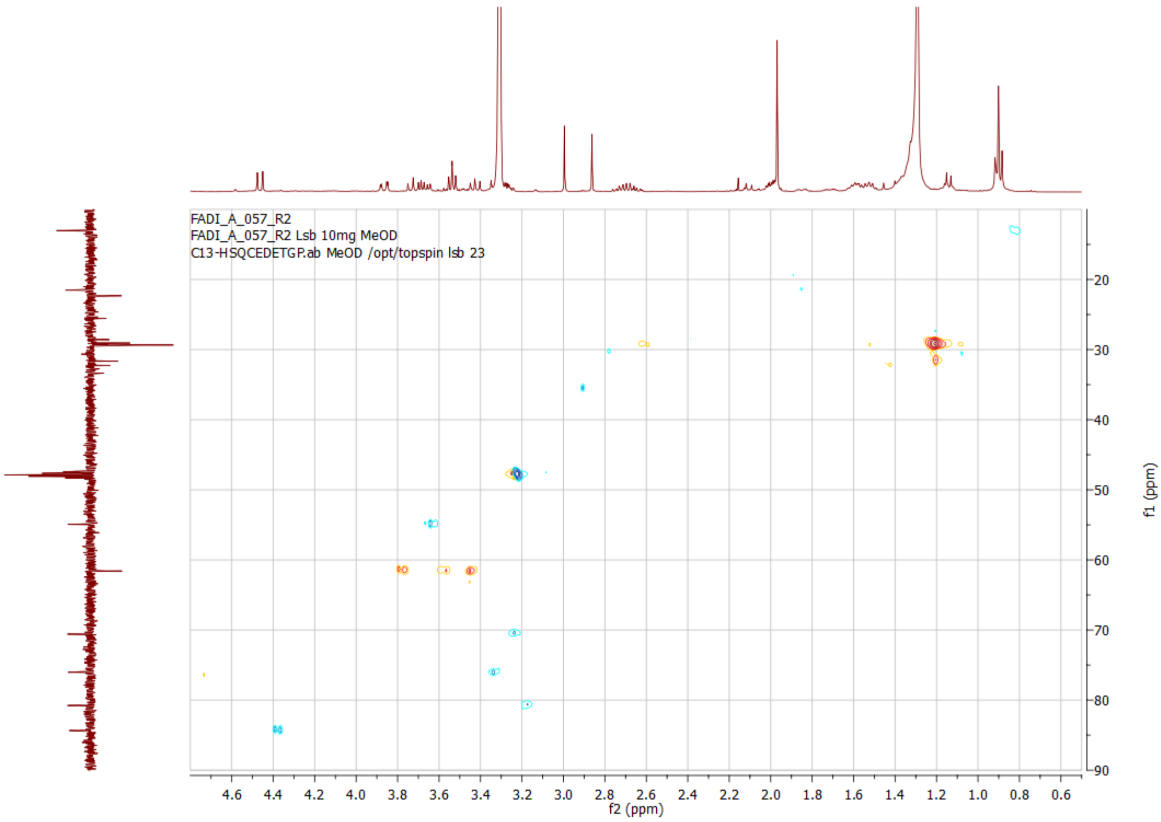

HSQC (CD_3OD) of compound 8

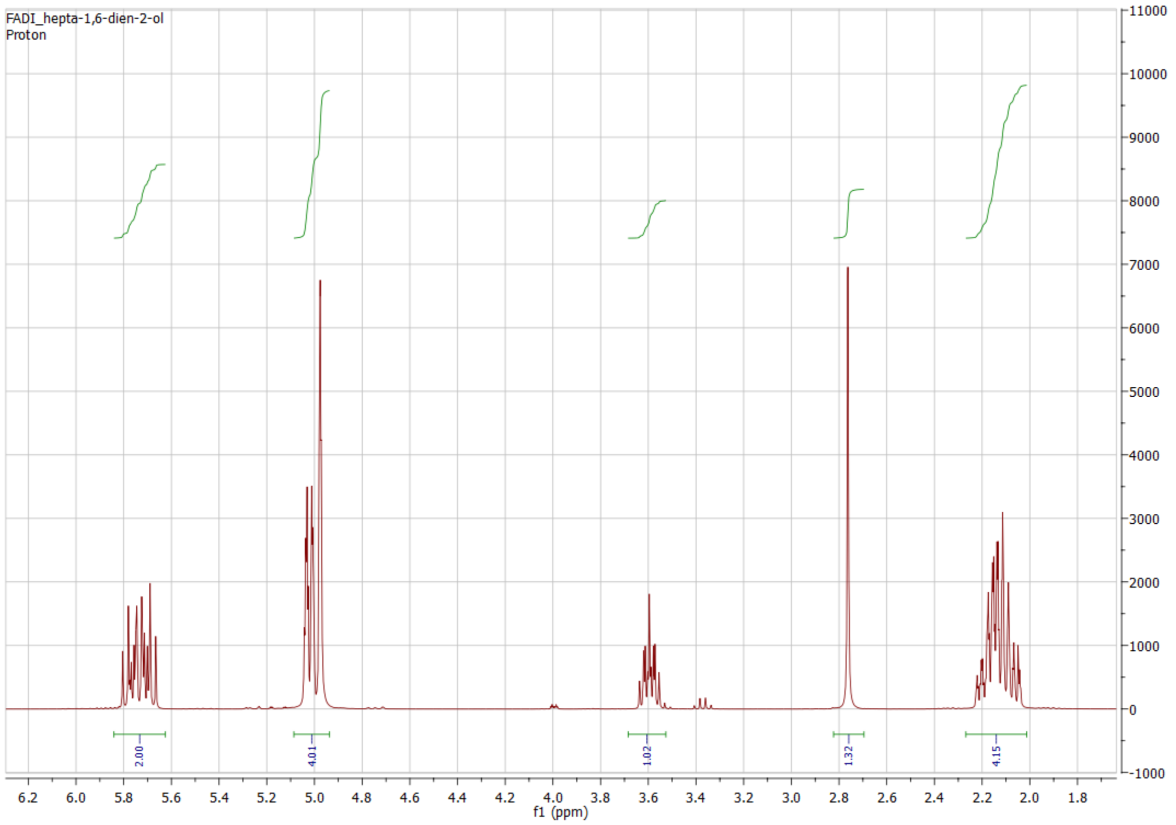

^{1}H NMR (300 MHz, $CDCl_3$) of compound 9

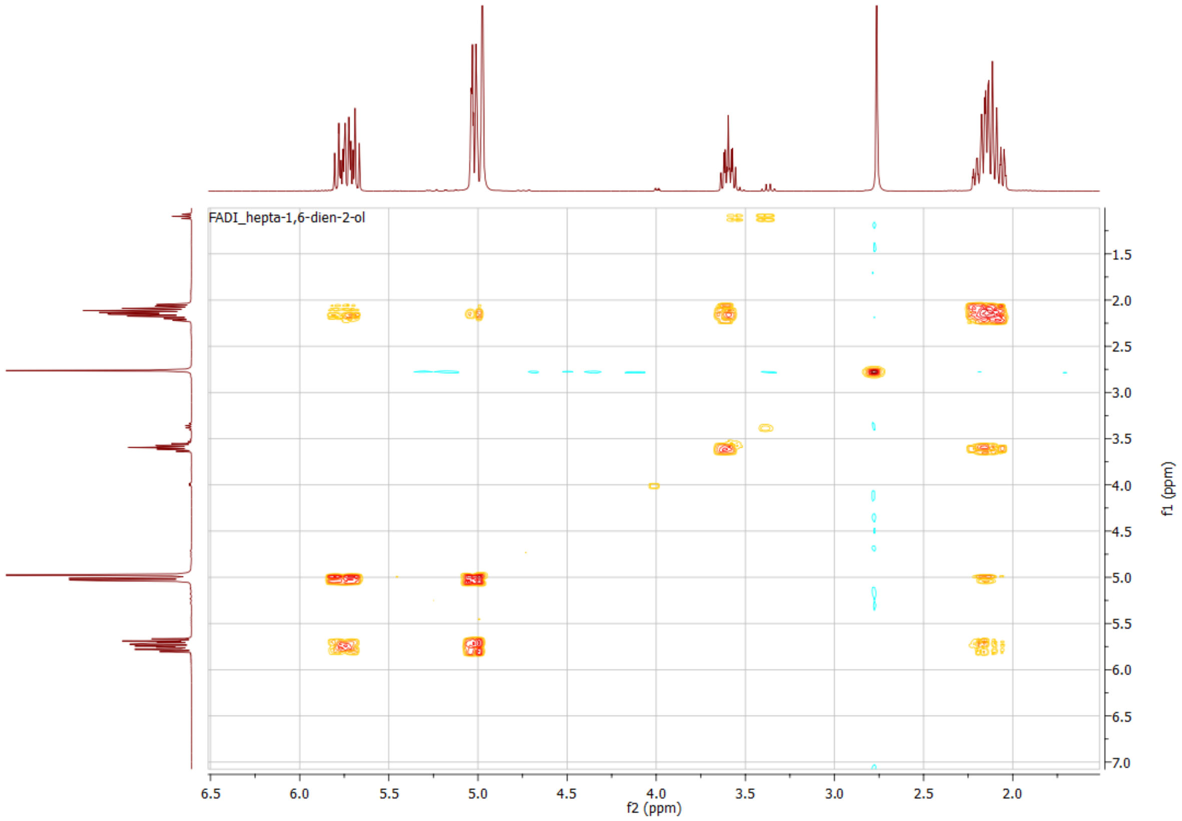

COSY (300 MHz, $CDCl_3$) of compound 9

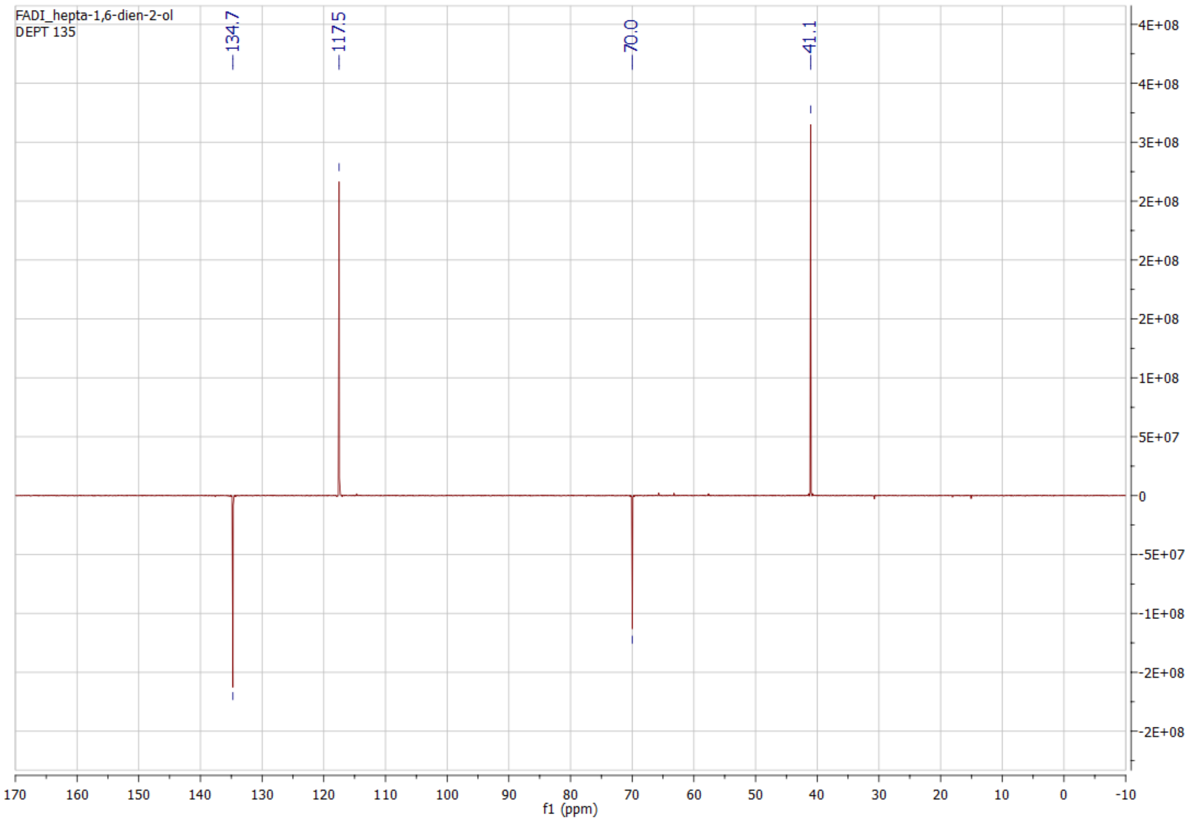

DEPT (75 MHz, $CDCl_3$) of compound 9
